# Supplementary figures and images for: ICAM-1 nanoclusters regulate hepatic epithelial cell polarity by leukocyte adhesion-independent control of apical actomyosin
Source: eLife. 2024 Apr 10;12:RP89261. doi: 10.7554/eLife.89261 (PMC11006420; doi:10.7554/eLife.89261)

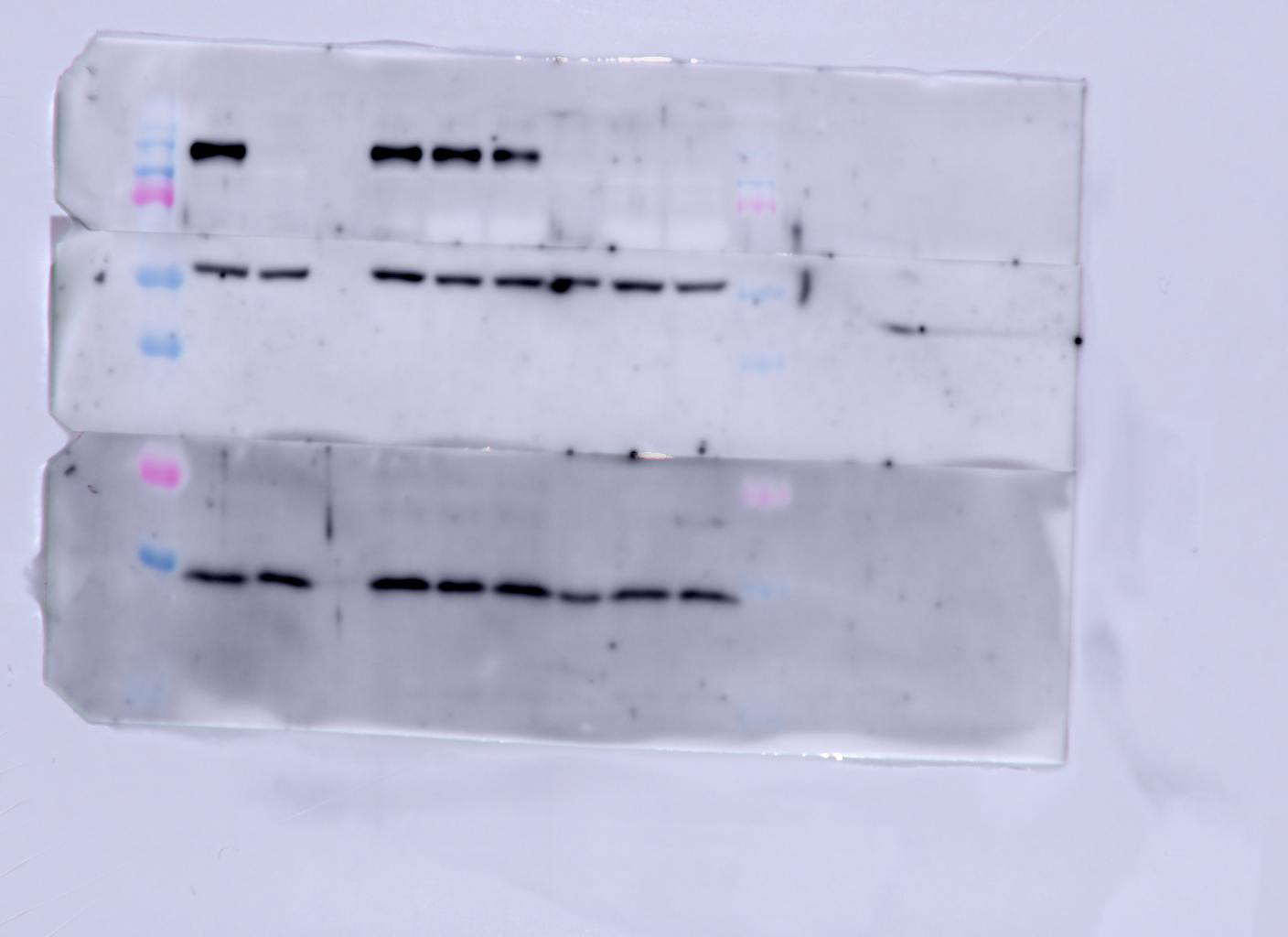

Supplement: Figure 1—source data 1. [file elife-89261-fig1-data1.zip › Figure 1 Source data 1.tif]

Fig. 1B

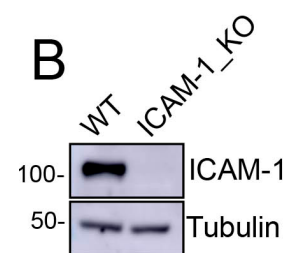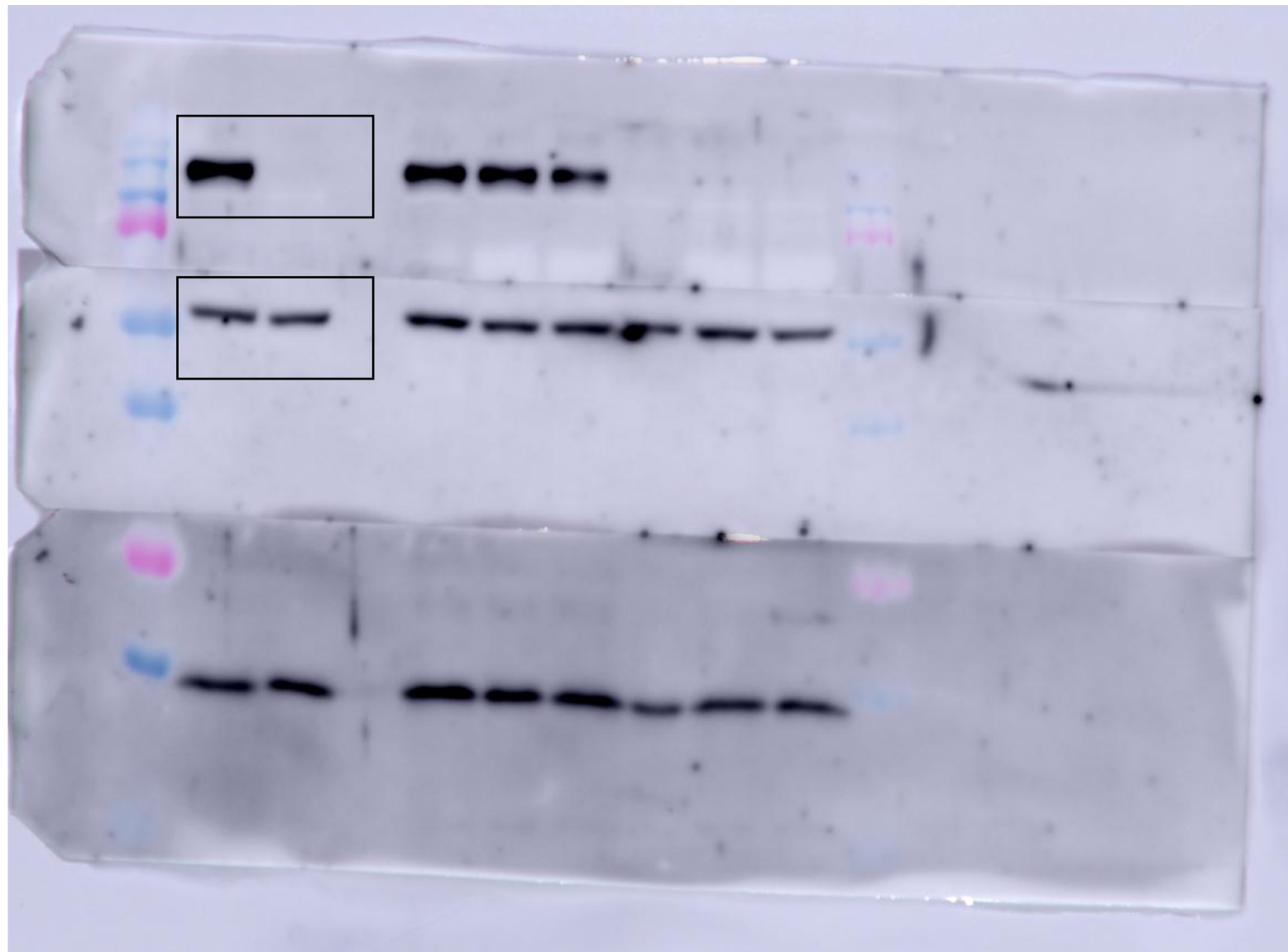

Supplement: Figure 1—source data 2. [file elife-89261-fig1-data2.zip › Figure 1 Source data 2.pdf]

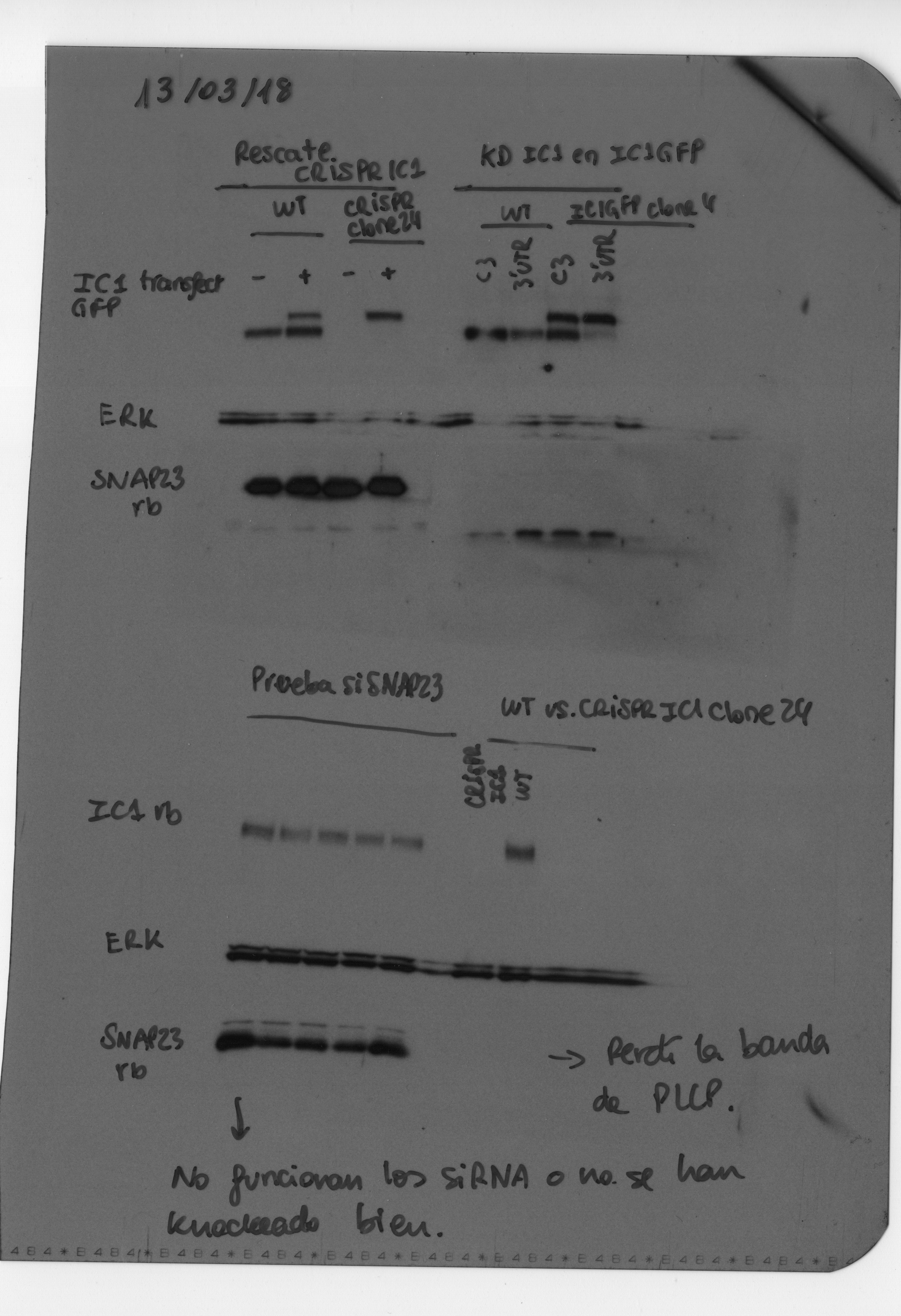

Supplement: Figure 1—source data 3. [file elife-89261-fig1-data3.zip › Figure 1 Source data 3.tif]

Fig. 1F

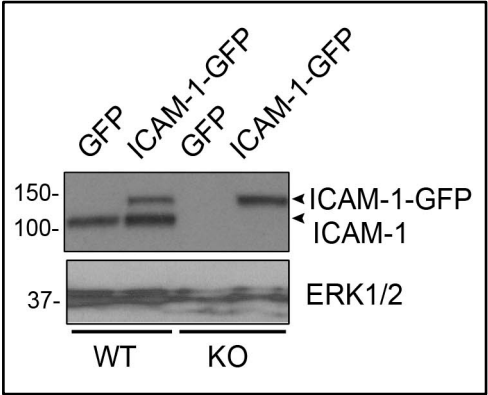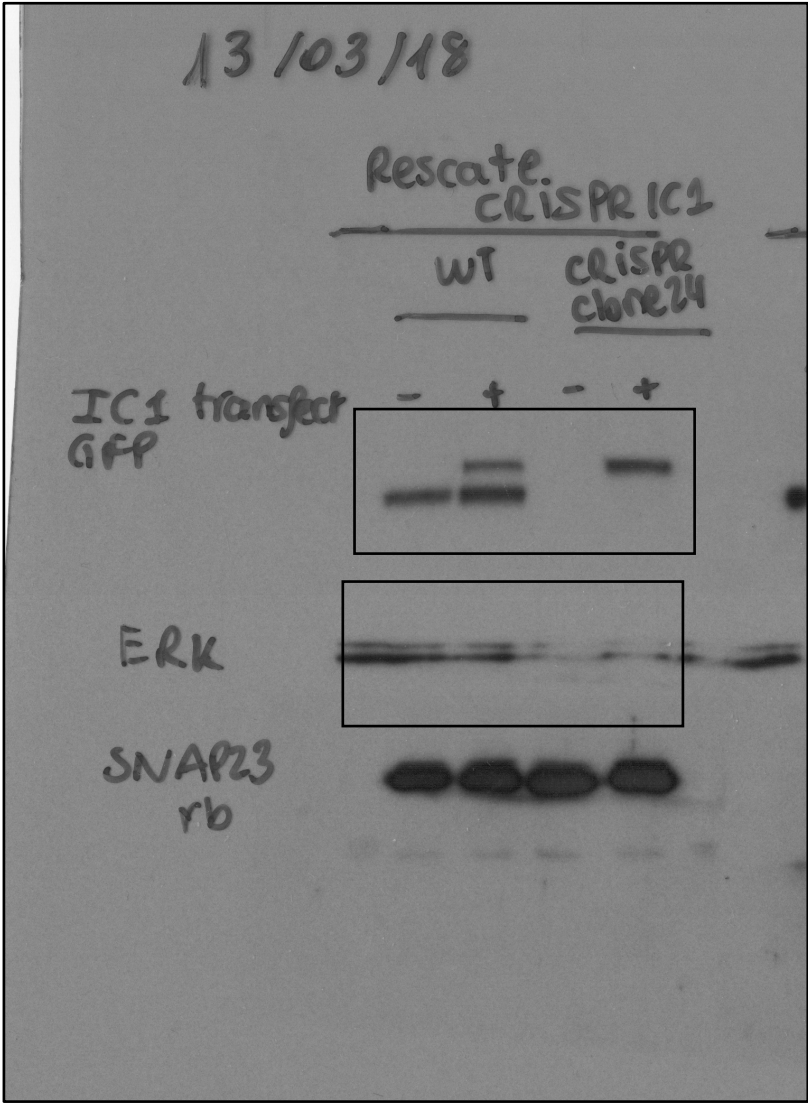

Supplement: Figure 1—source data 4. [file elife-89261-fig1-data4.zip › Figure 1 Source data 4.pdf]

Fig1-figure supplement 1F  
(from screening several siRNA)

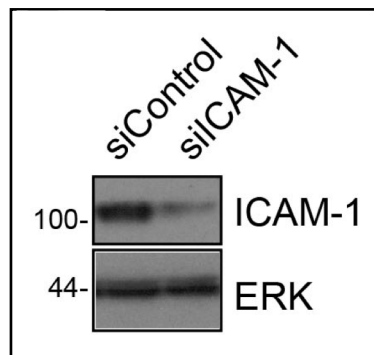

exposure for ICAM-1

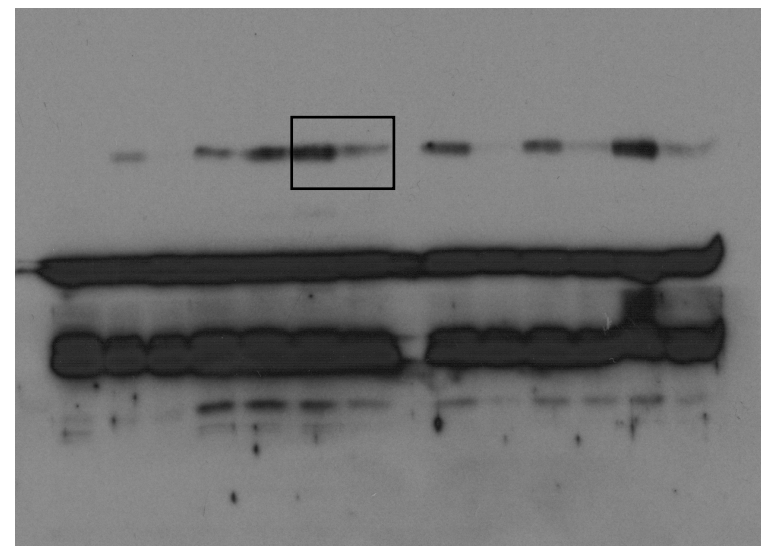

exposure for ERK

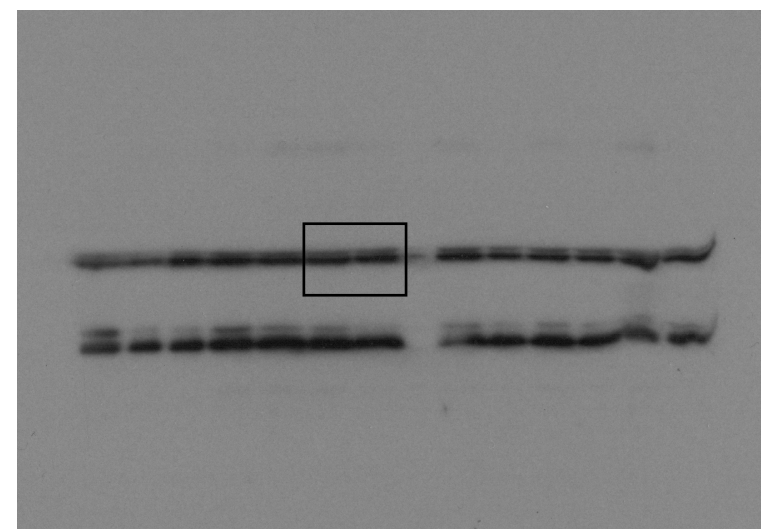

Supplement: Figure 1—figure supplement 1—source data 2. [file elife-89261-fig1-figsupp1-data2.zip › Figure 1-supplement figure 1-source data 2.pdf]

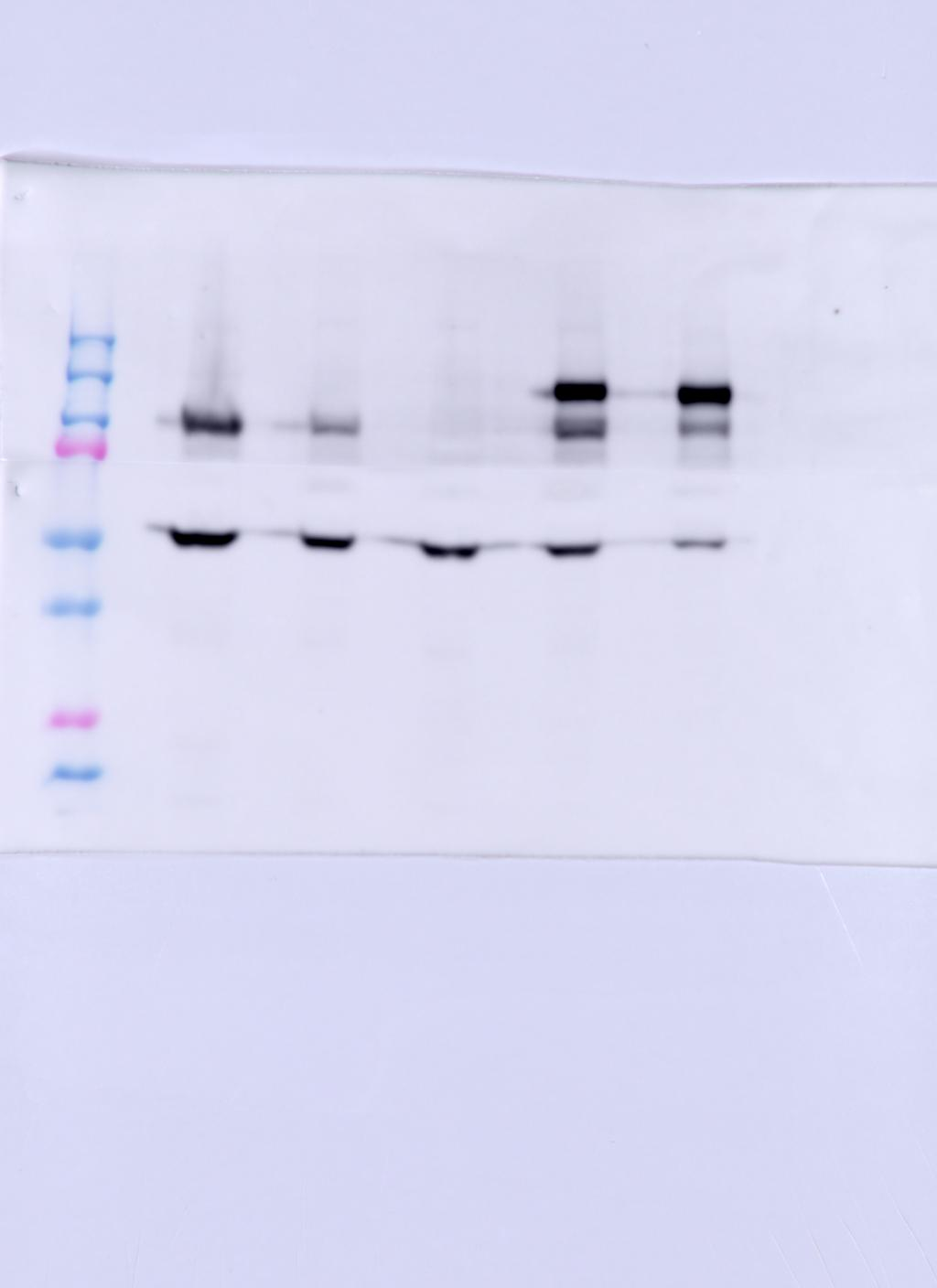

Supplement: Figure 2—figure supplement 1—source data 1. [file elife-89261-fig2-figsupp1-data1.zip › Figure 2-supplement figure 1-source data 1.tiff]

Fig2-figure supplement 1B

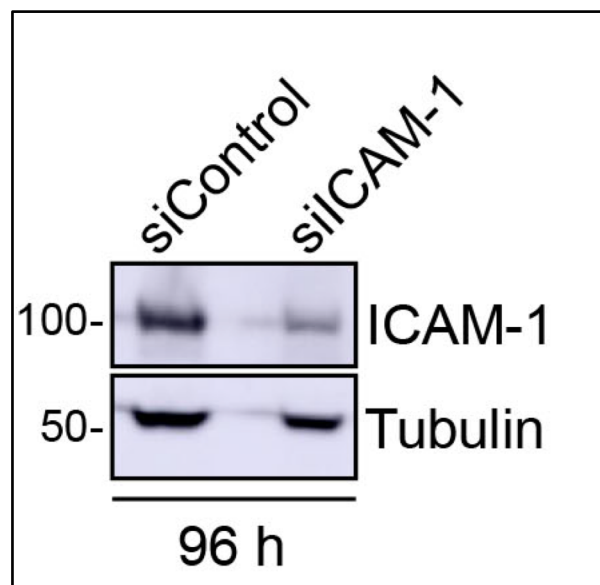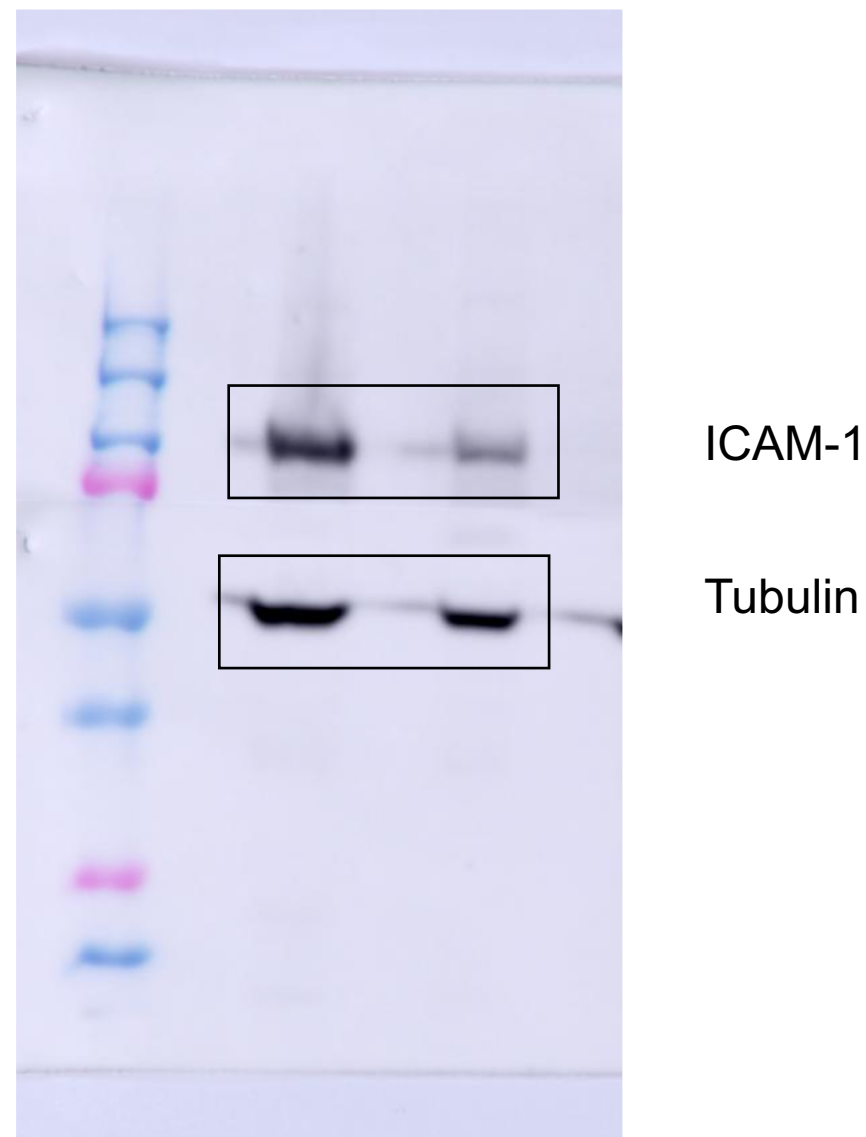

Supplement: Figure 2—figure supplement 1—source data 2. [file elife-89261-fig2-figsupp1-data2.zip › Figure 2-supplement figure 1-source data 2.pdf]

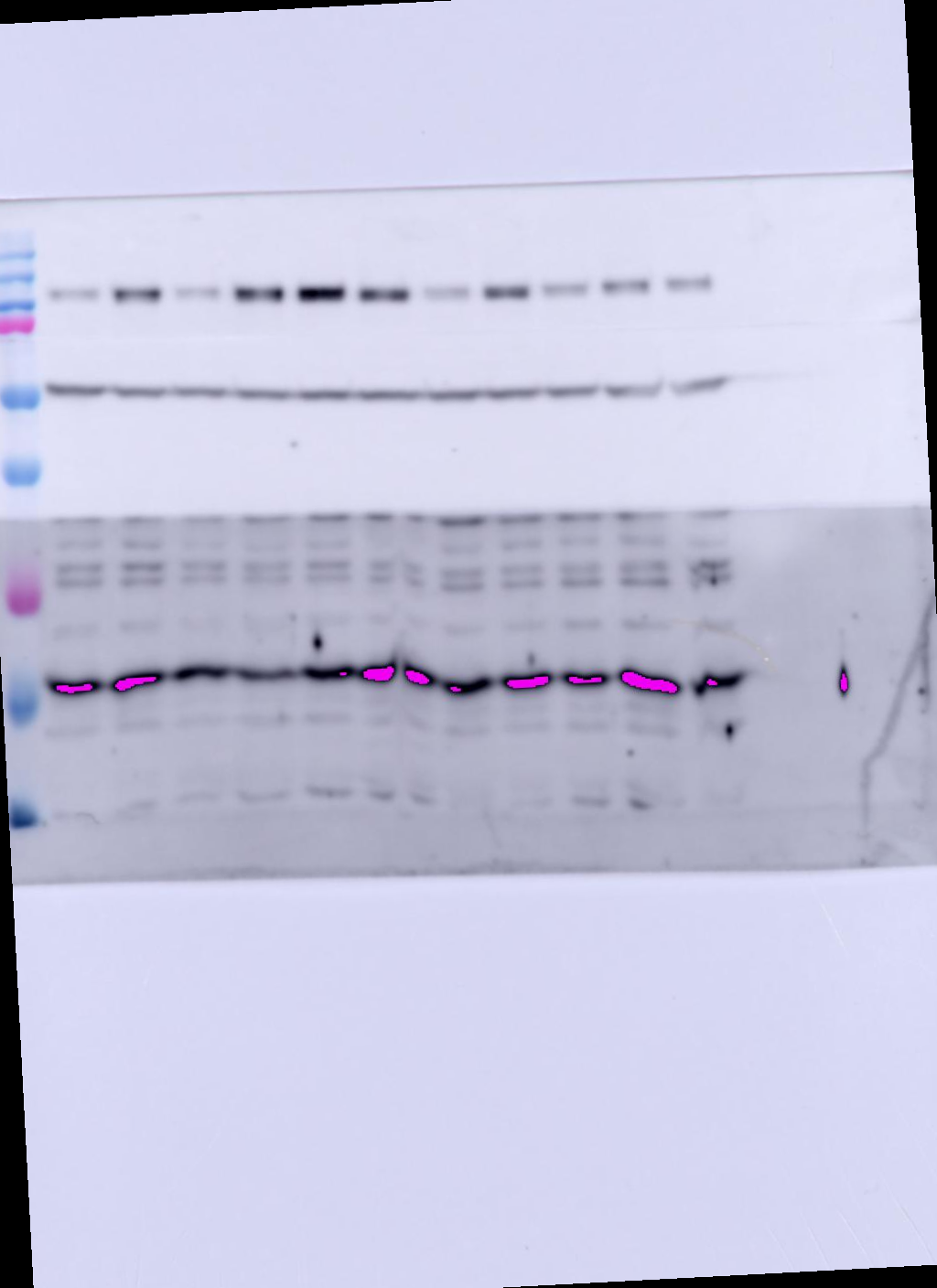

Supplement: Figure 3—source data 1. [file elife-89261-fig3-data1.zip › Figure 3 Source data 1.tiff]

Fig. 3A

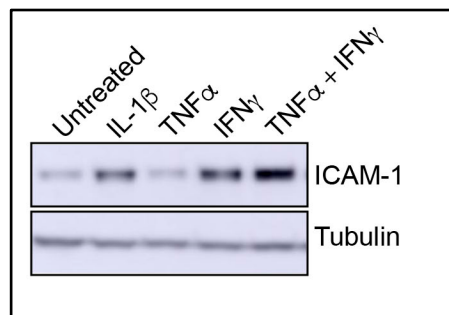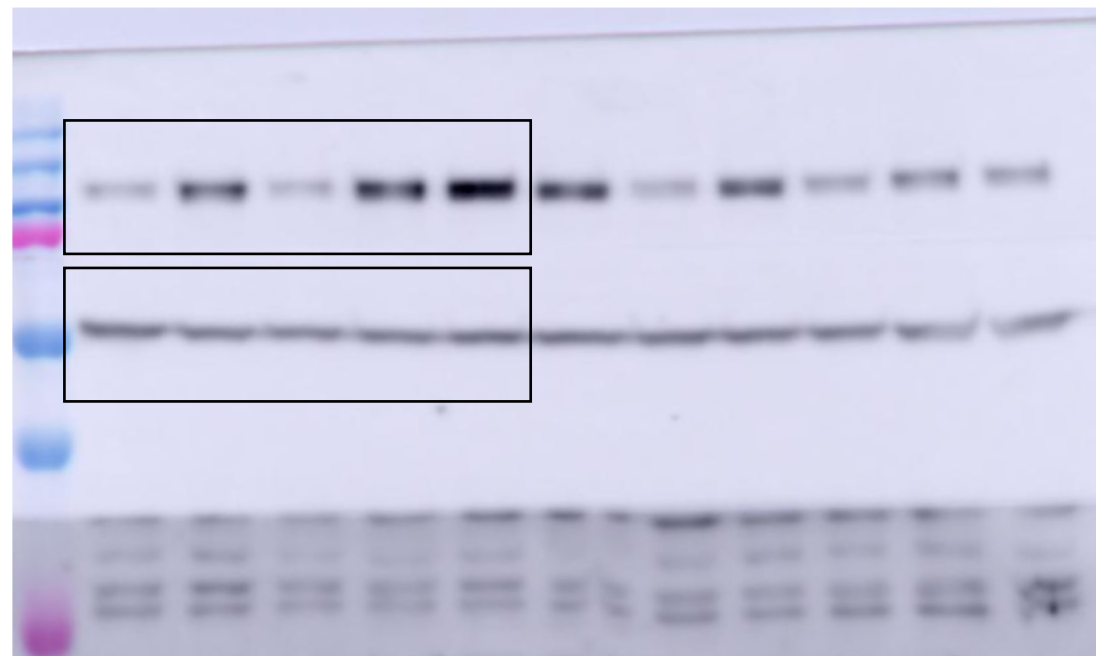

Supplement: Figure 3—source data 2. [file elife-89261-fig3-data2.zip › Figure 3 Source data 2.pdf]

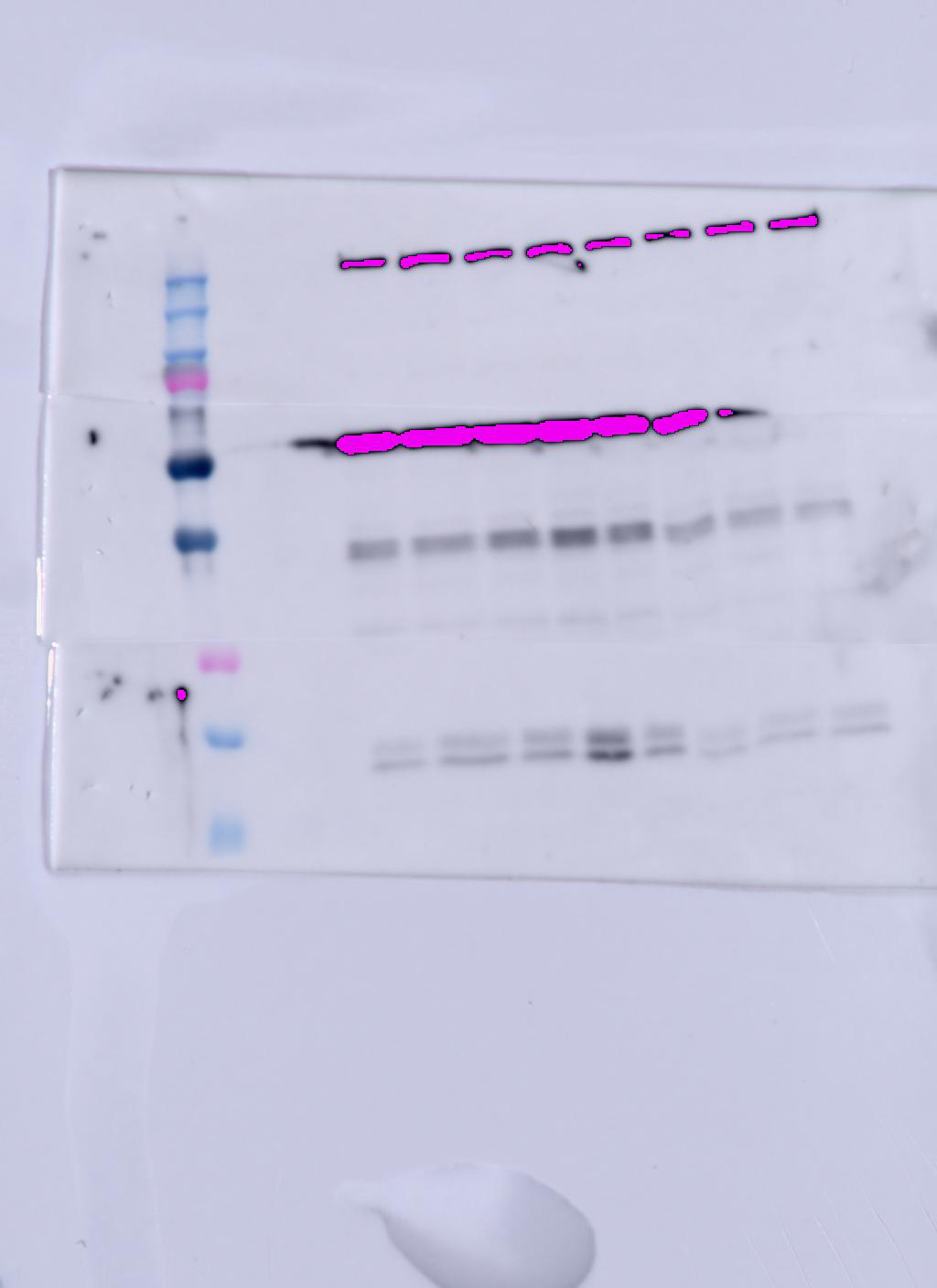

Supplement: Figure 5—source data 1. [file elife-89261-fig5-data1.zip › Figure 5 Source data 1.tif]

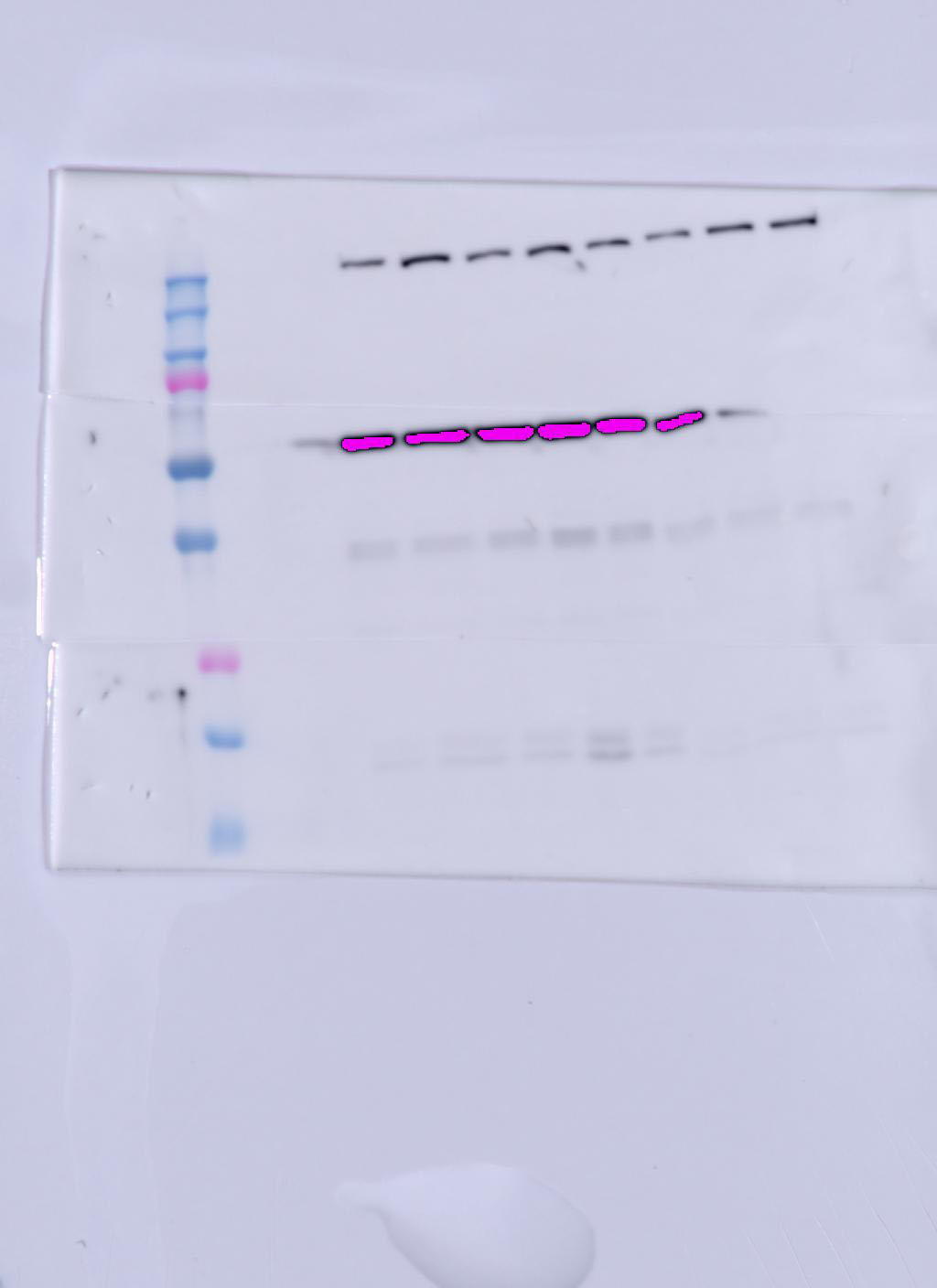

Supplement: Figure 5—source data 2. [file elife-89261-fig5-data2.zip › Figure 5 Source data 2.tif]

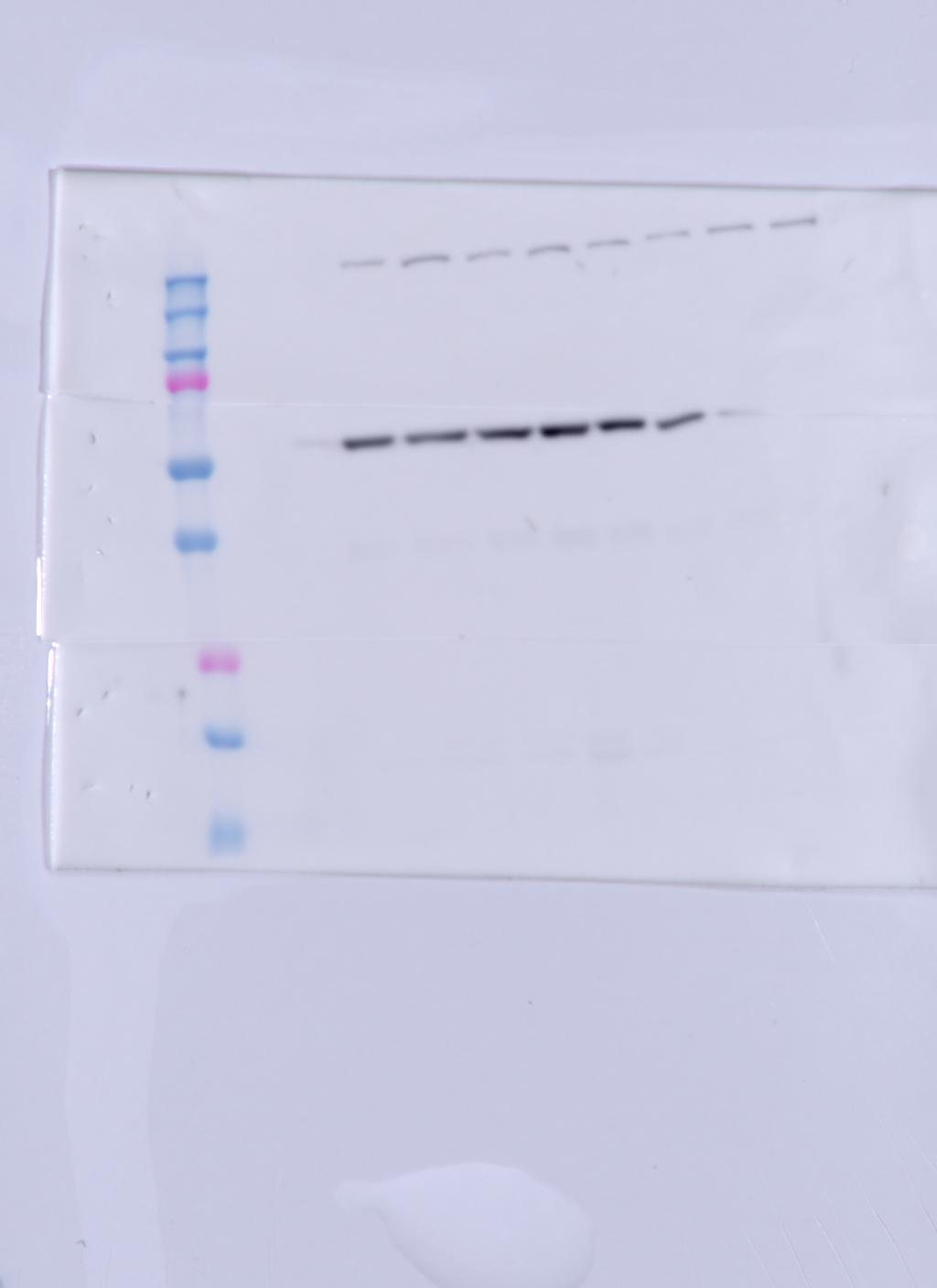

Supplement: Figure 5—source data 3. [file elife-89261-fig5-data3.zip › Figure 5 Source data 3.tif]

Fig. 5E

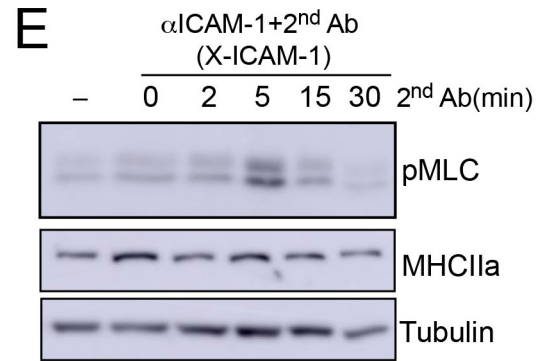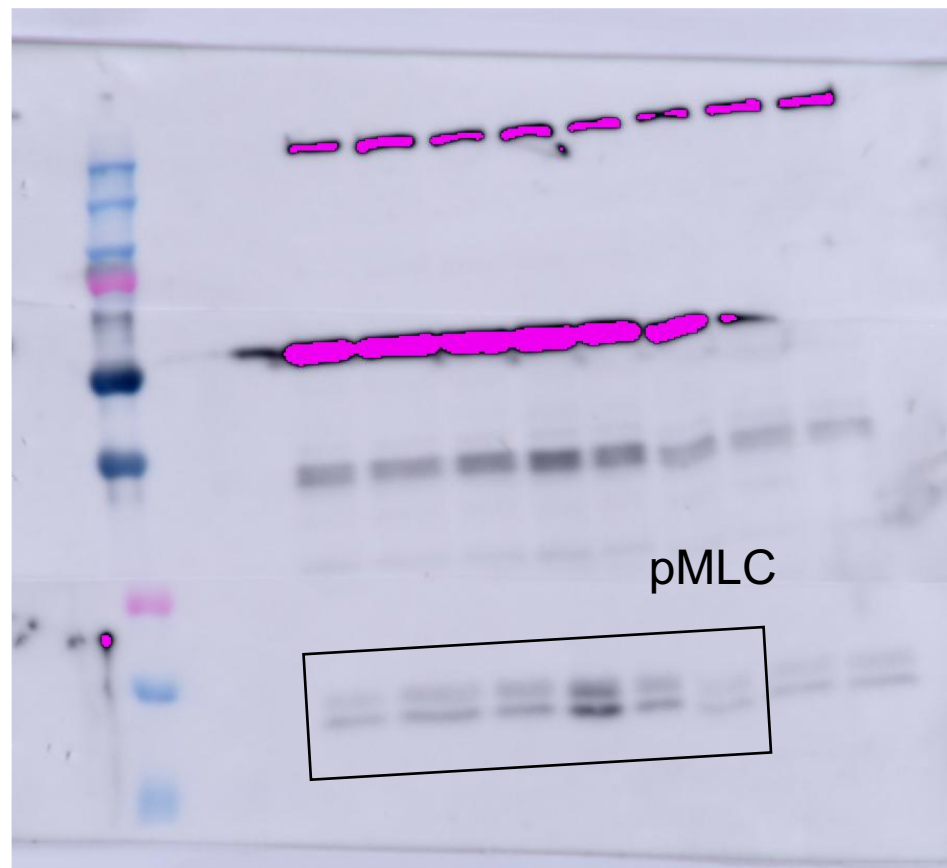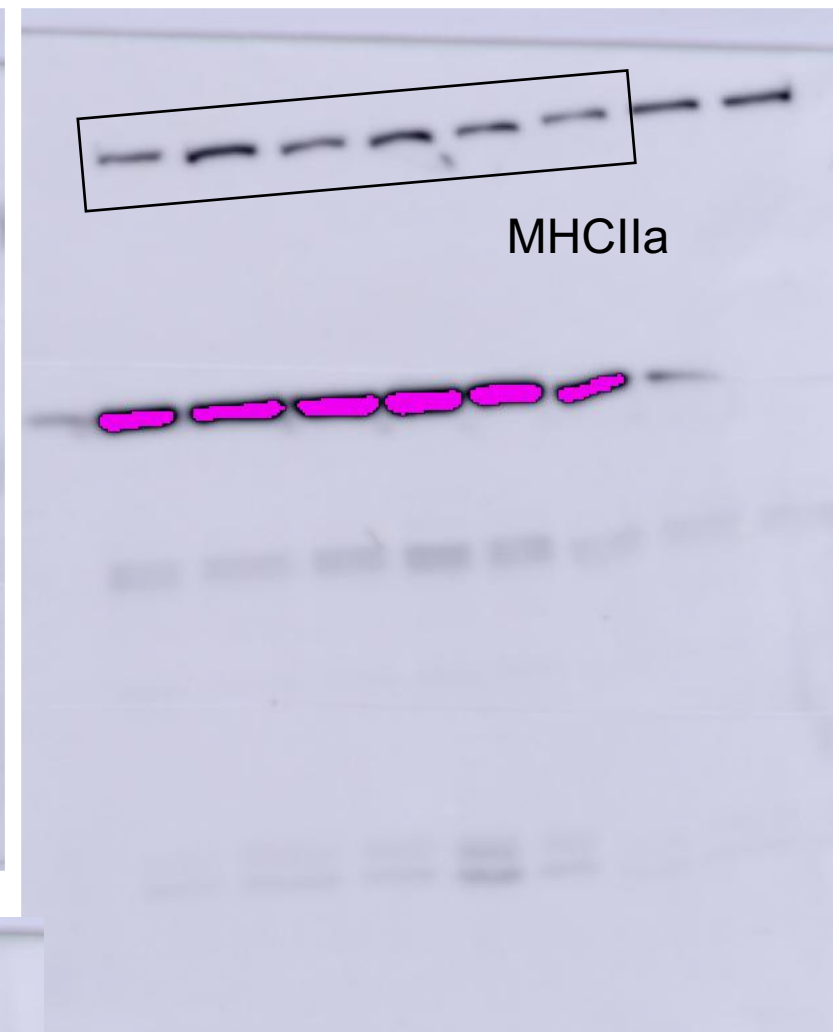

Supplement: Figure 5—source data 4. [file elife-89261-fig5-data4.zip › Figure 5 Source data 4.pdf]

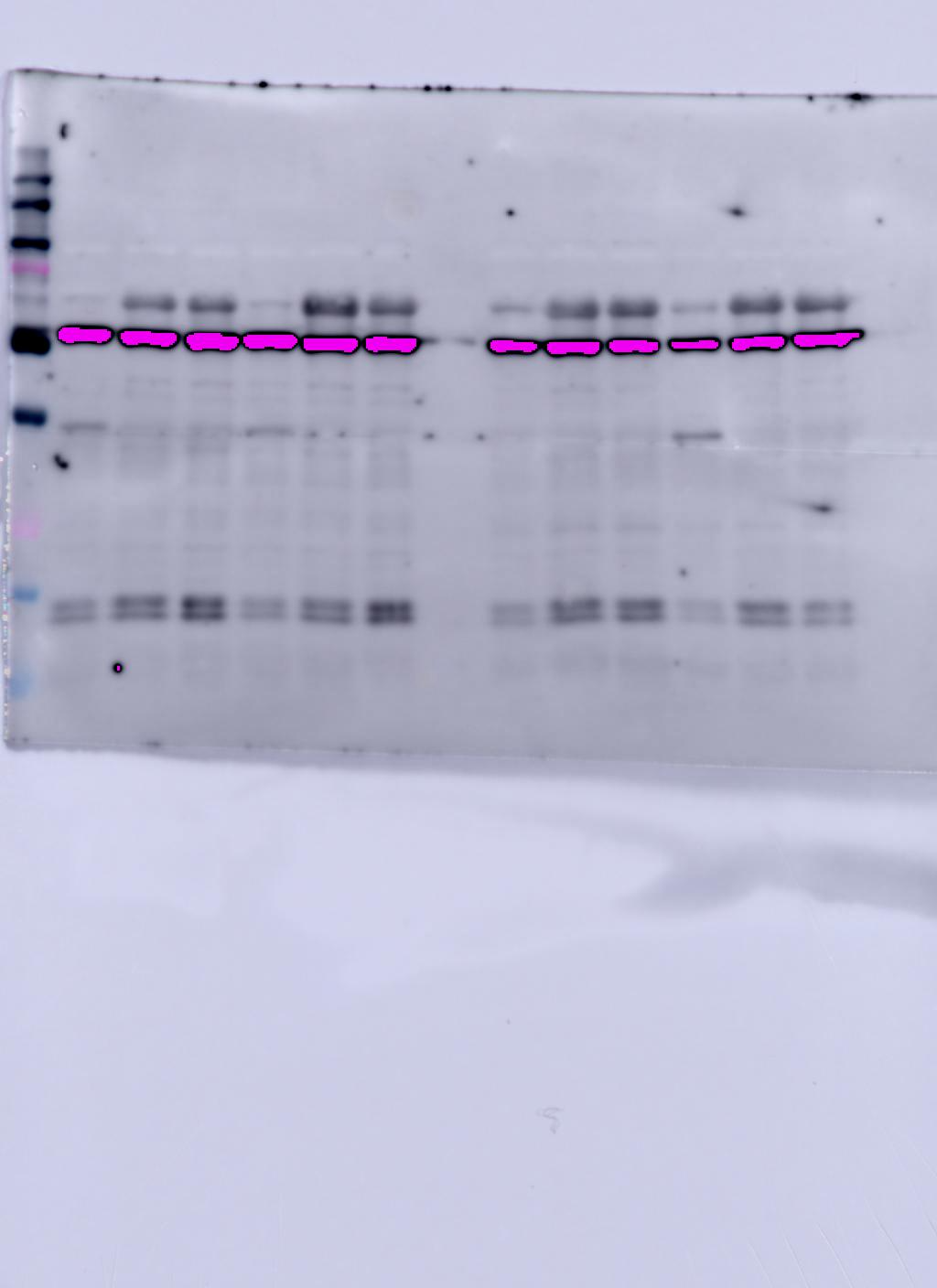

Supplement: Figure 5—figure supplement 1—source data 1. [file elife-89261-fig5-figsupp1-data1.zip › Figure 5-supplement figure 1-source data 1.tiff]

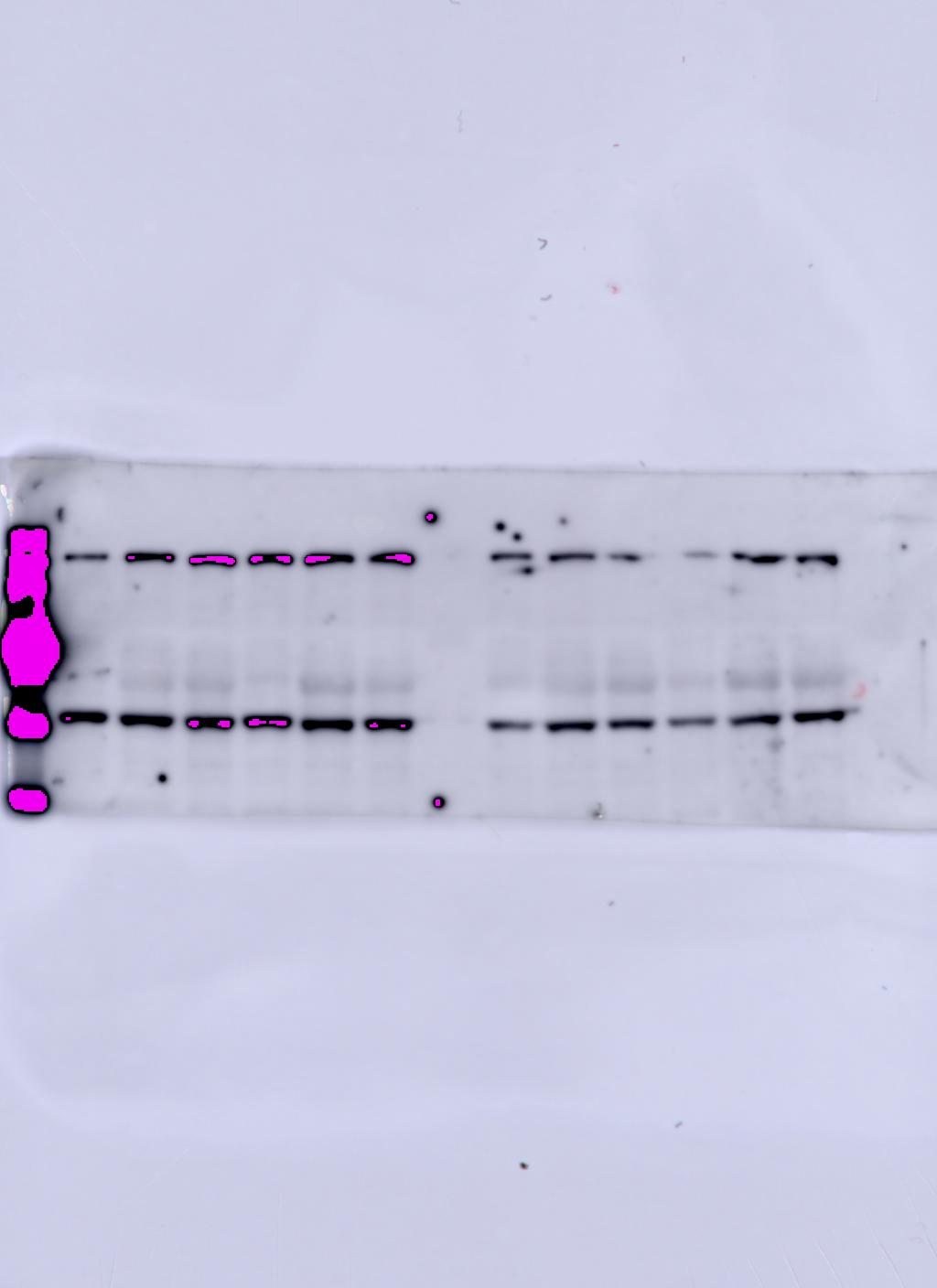

Supplement: Figure 5—figure supplement 1—source data 2. — Exposure corresponding to ICAM-1_KO cells. [file elife-89261-fig5-figsupp1-data2.zip › Figure 5-supplement figure 1-source data 2.tiff]

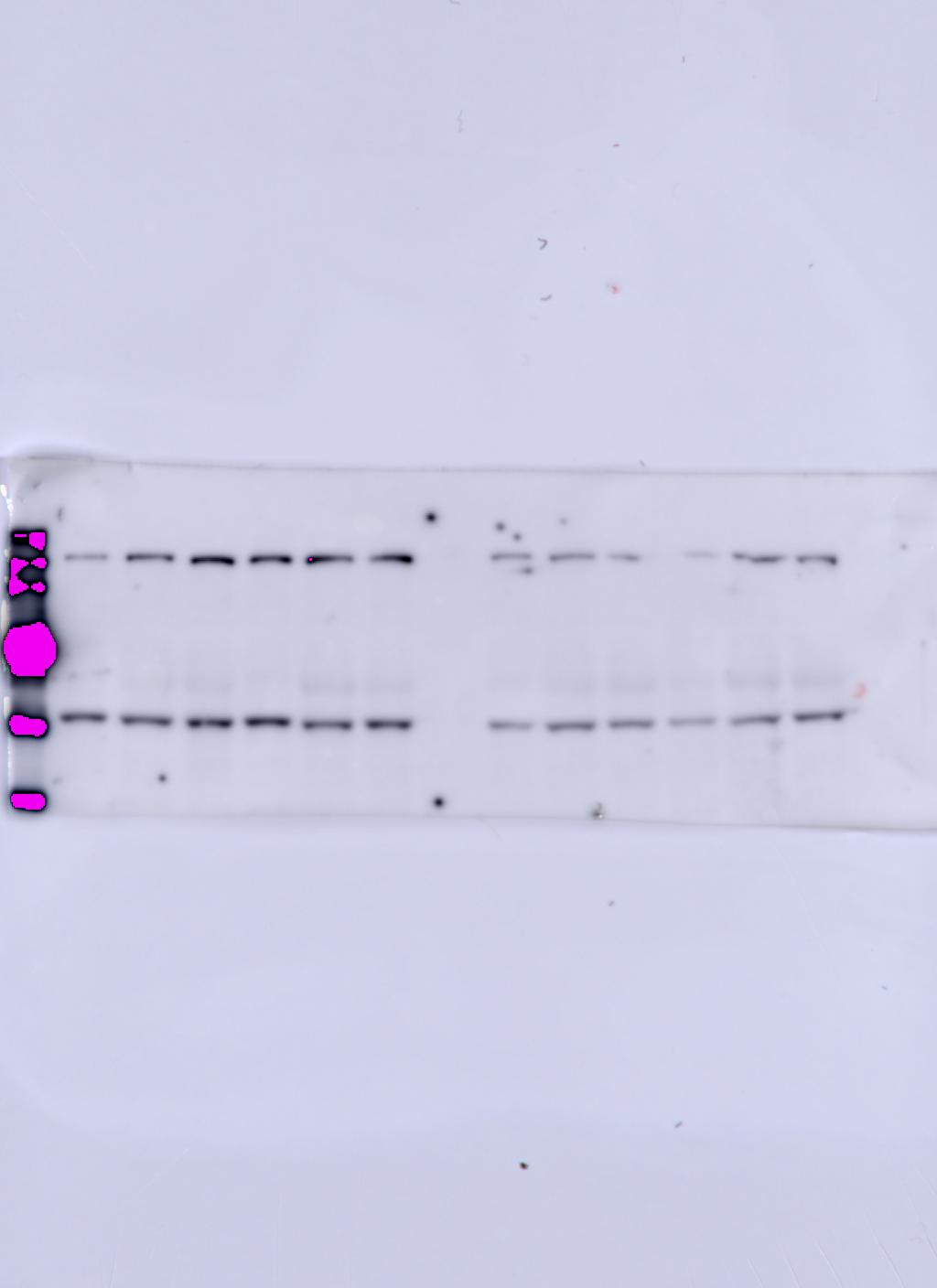

Supplement: Figure 5—figure supplement 1—source data 3. — Exposure corresponding to WT cells. [file elife-89261-fig5-figsupp1-data3.zip › Figure 5-supplement figure 1-source data 3.tiff]

Fig5-figure supplement 1C

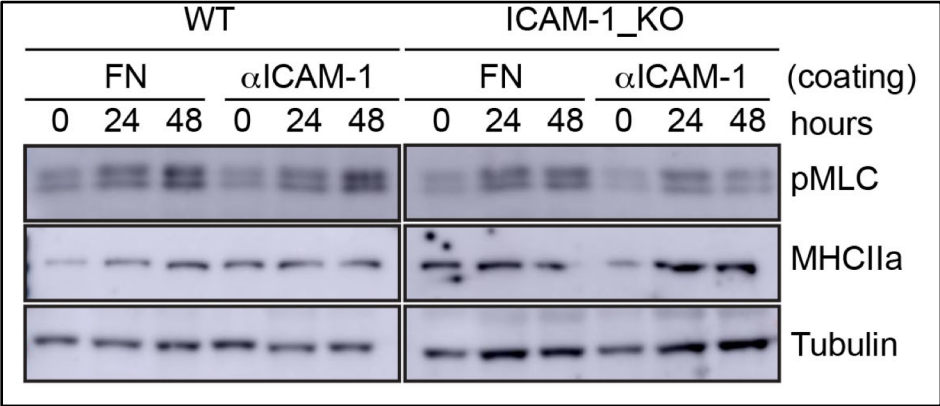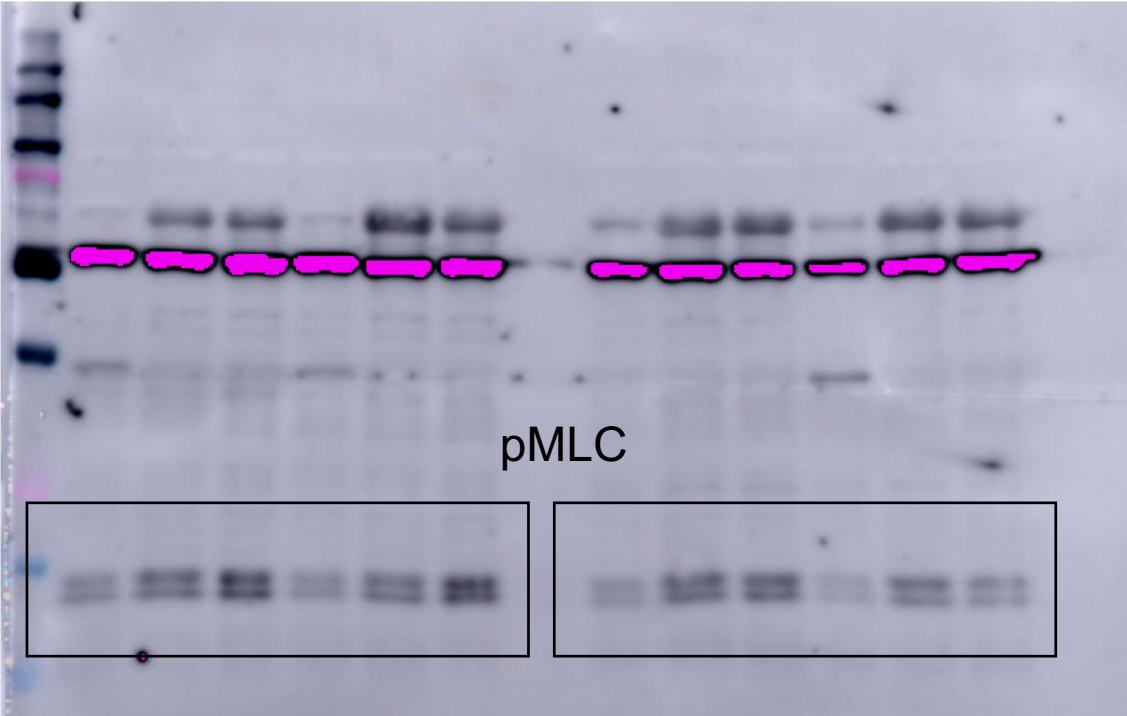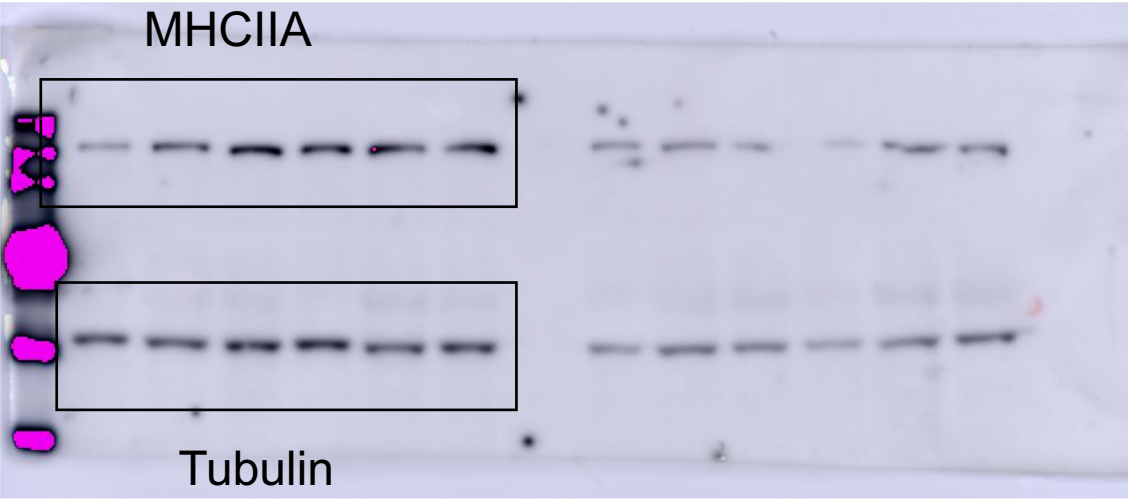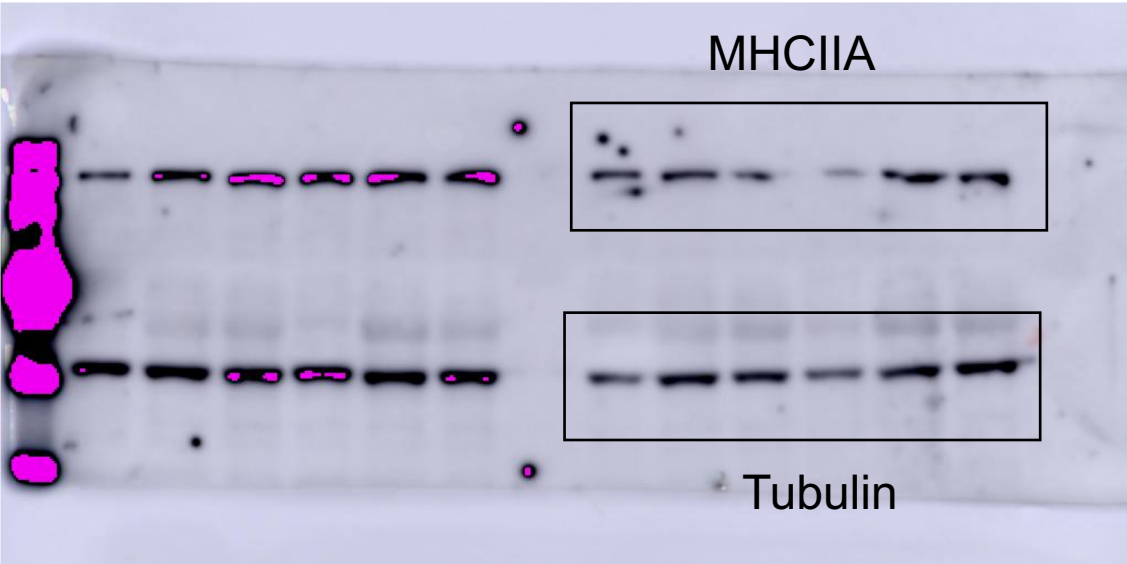

Supplement: Figure 5—figure supplement 1—source data 4. [file elife-89261-fig5-figsupp1-data4.zip › Figure 5-supplement figure 1-source data 4.pdf]

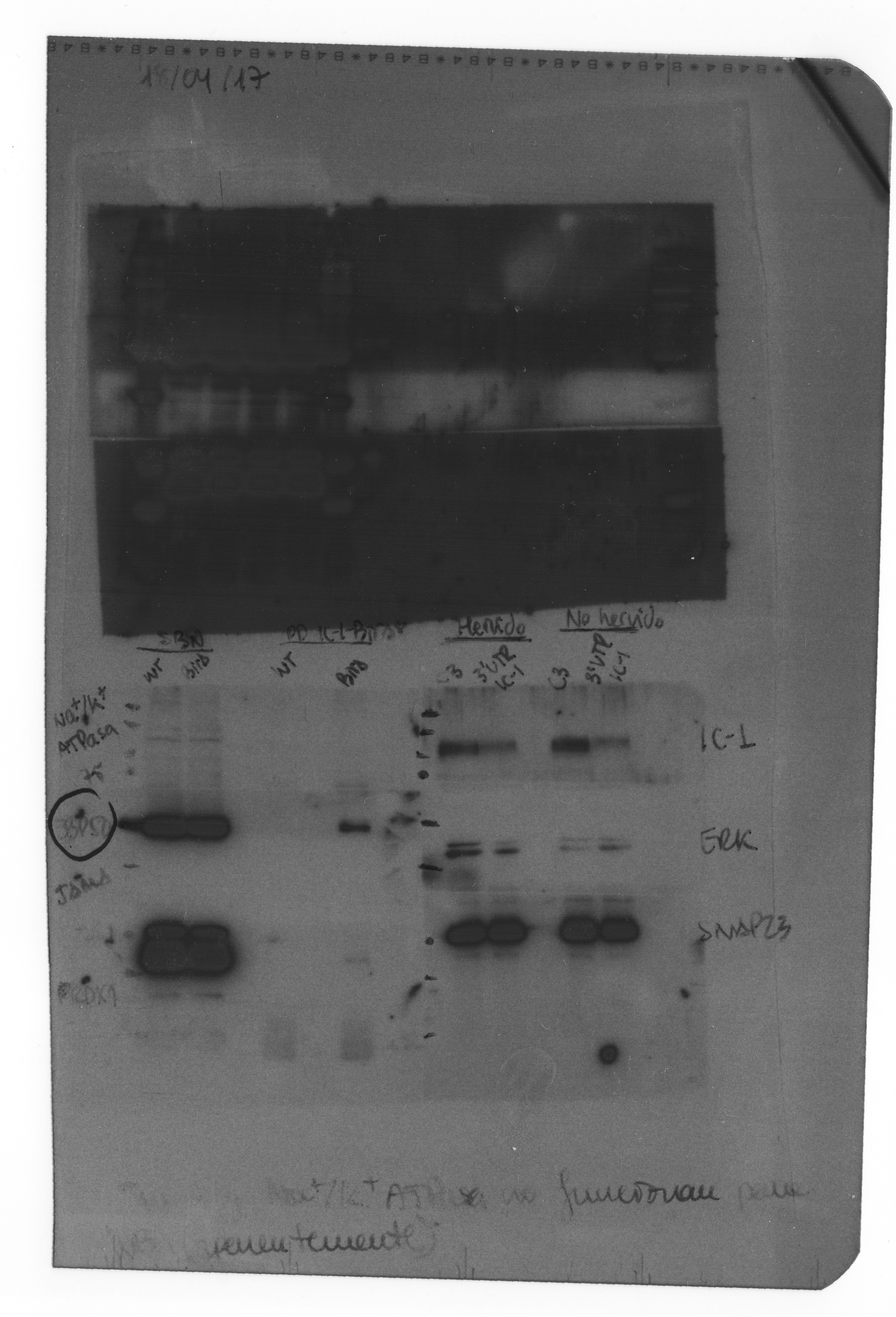

Supplement: Figure 6—source data 1. [file elife-89261-fig6-data1.zip › Figure 6 Source data 1.tif]

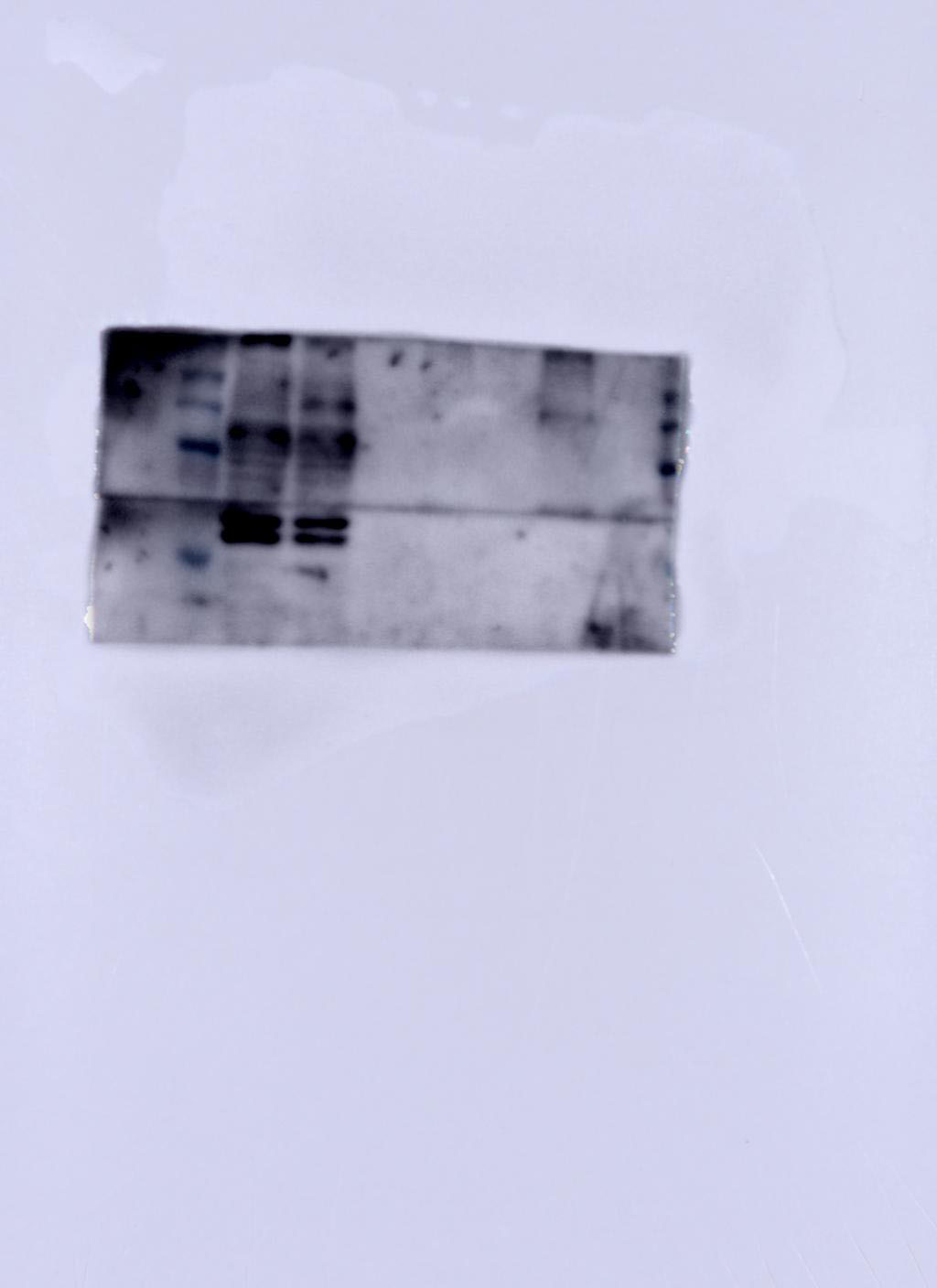

Supplement: Figure 6—source data 2. [file elife-89261-fig6-data2.zip › Figure 6 Source data 2.tif]

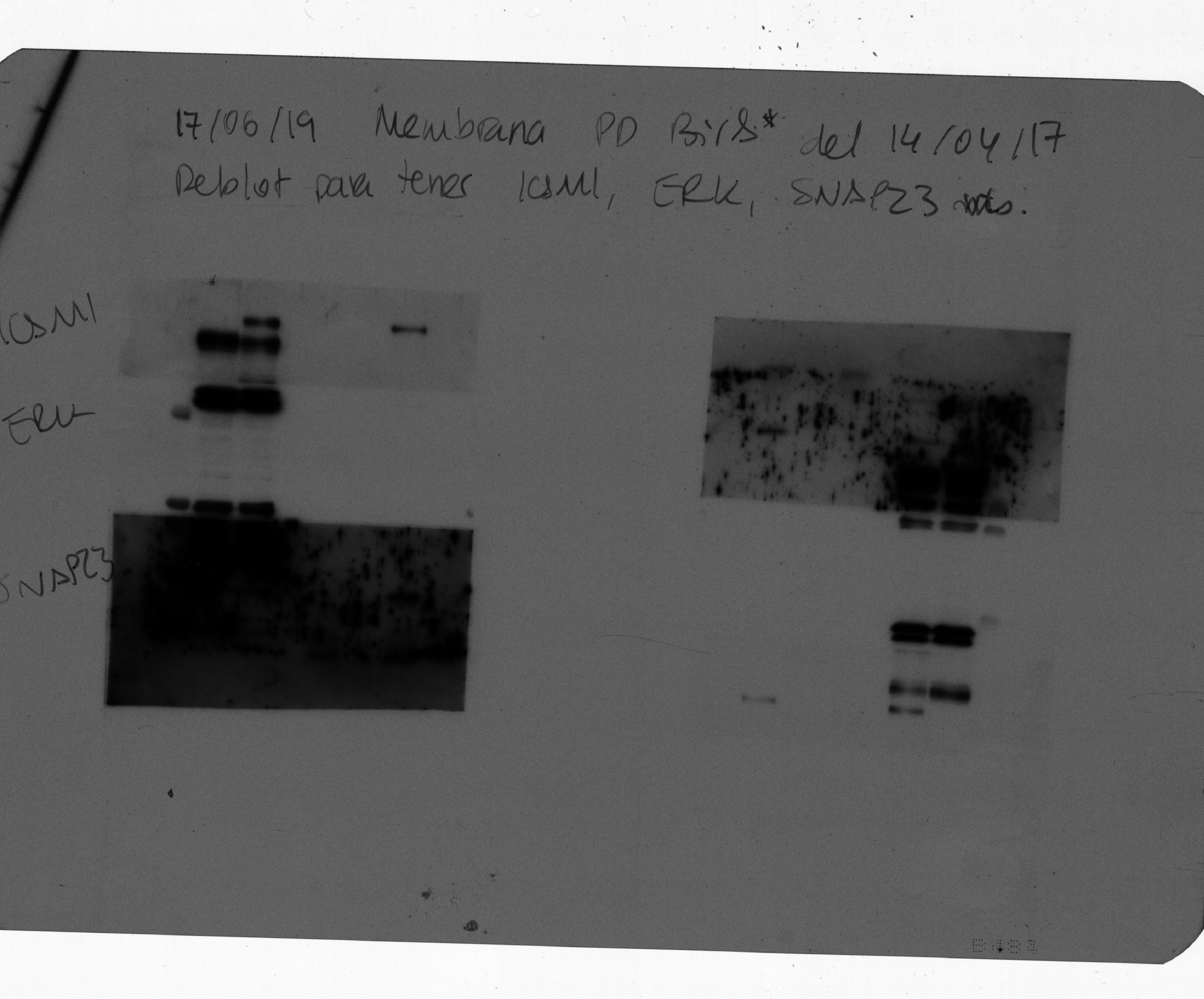

Supplement: Figure 6—source data 3. [file elife-89261-fig6-data3.zip › Figure 6 Source data 3.tif]

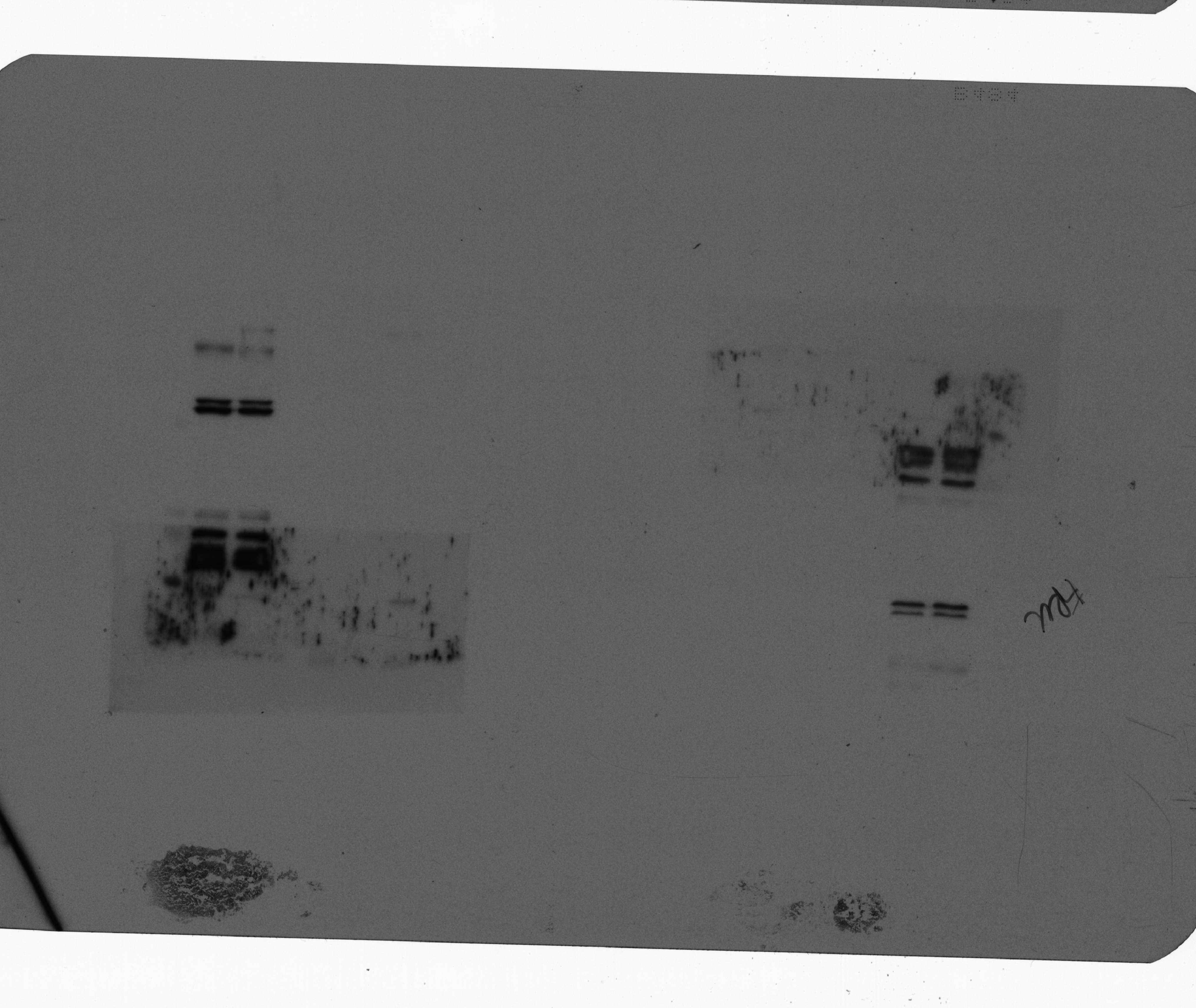

Supplement: Figure 6—source data 4. [file elife-89261-fig6-data4.zip › Figure 6 source data 4.tif]

Fig. 6A

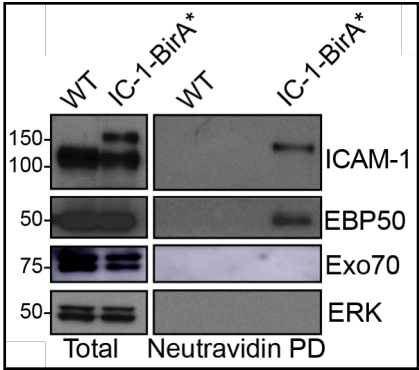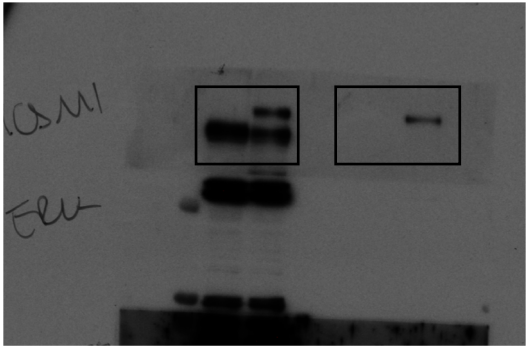

ICAM-1

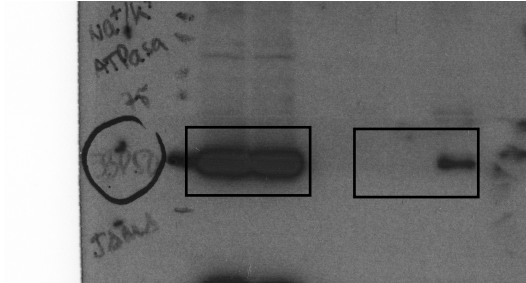

EBP50

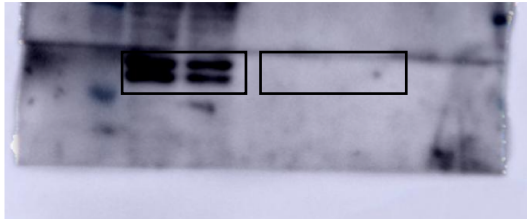

EXO70

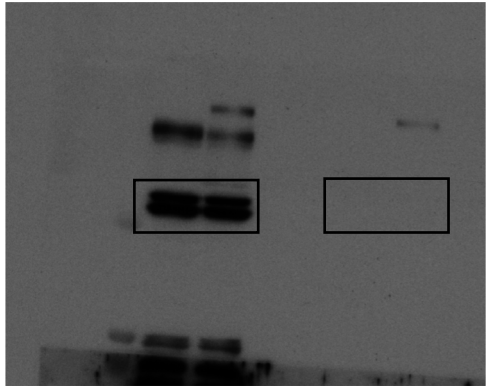

ERK

Supplement: Figure 6—source data 5. [file elife-89261-fig6-data5.zip › Figure 6 Source data 5.pdf]

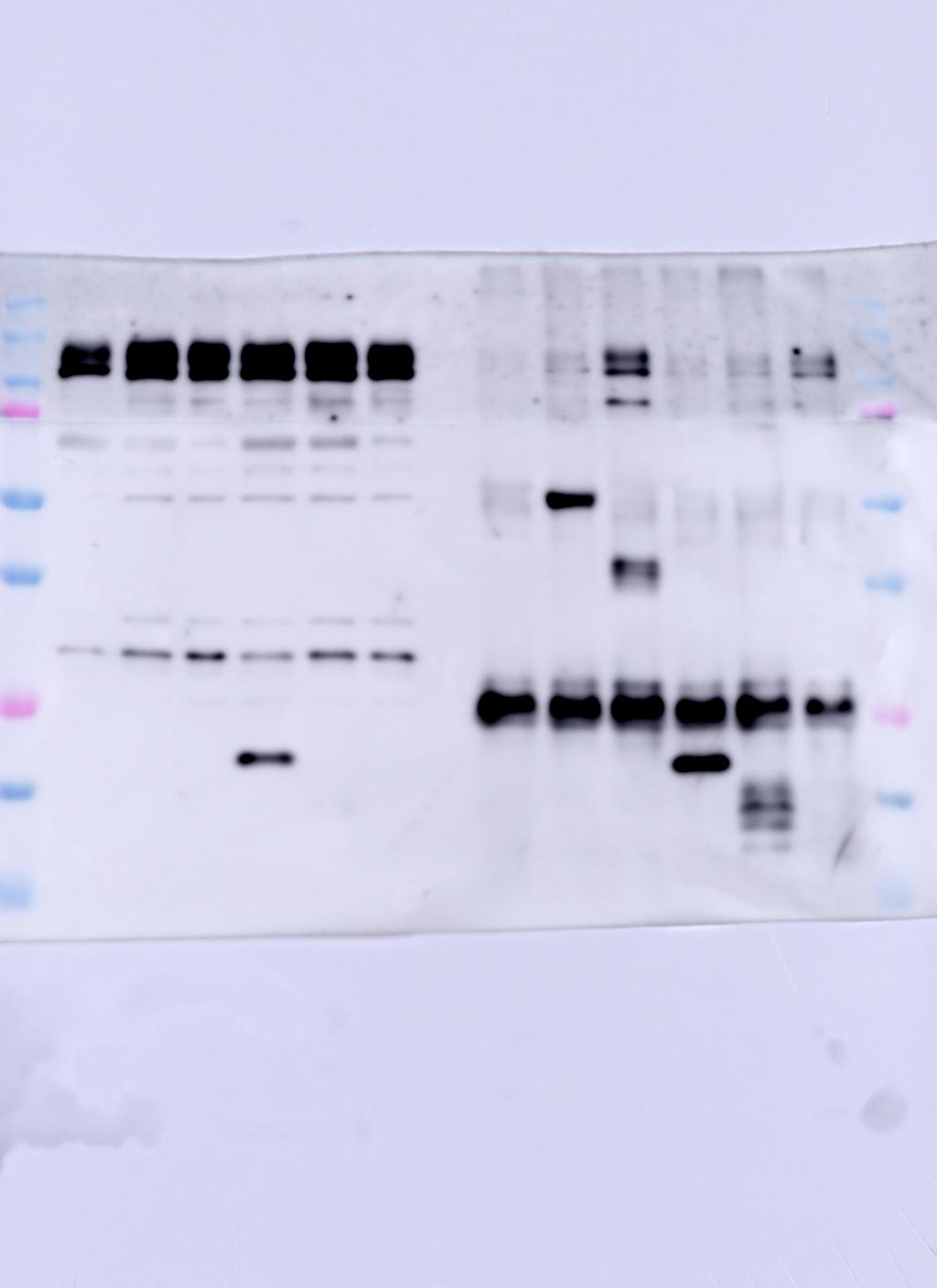

Supplement: Figure 6—source data 6. [file elife-89261-fig6-data6.zip › Figure 6 Source data 6.tiff]

Fig. 6E

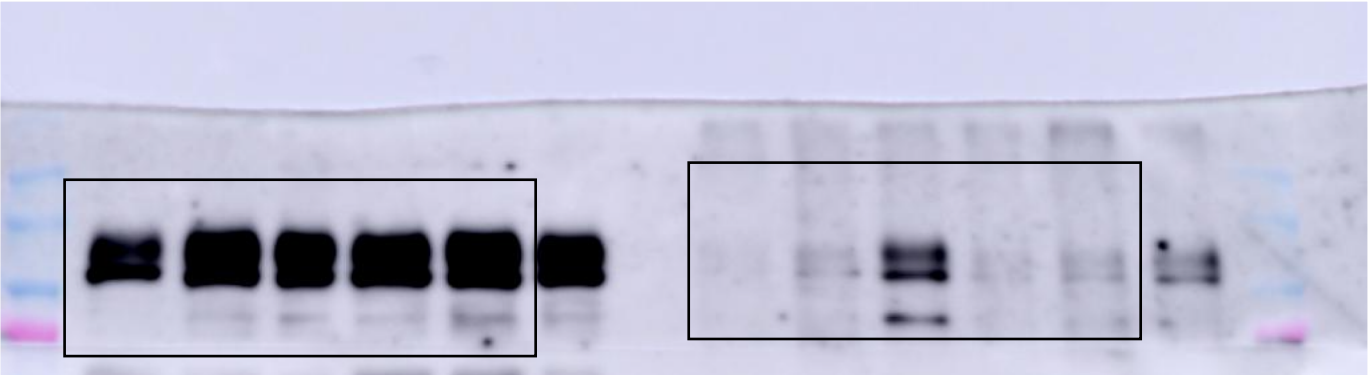

ICAM-1

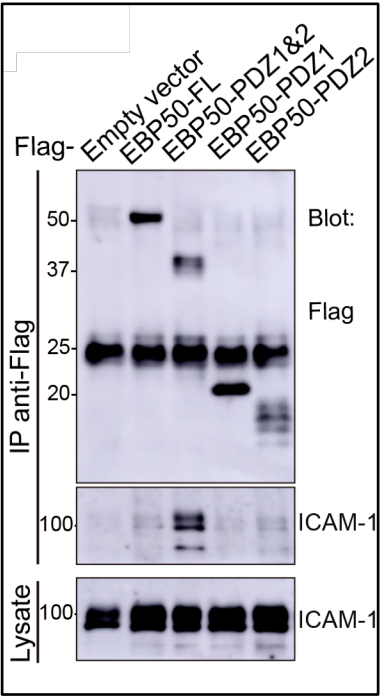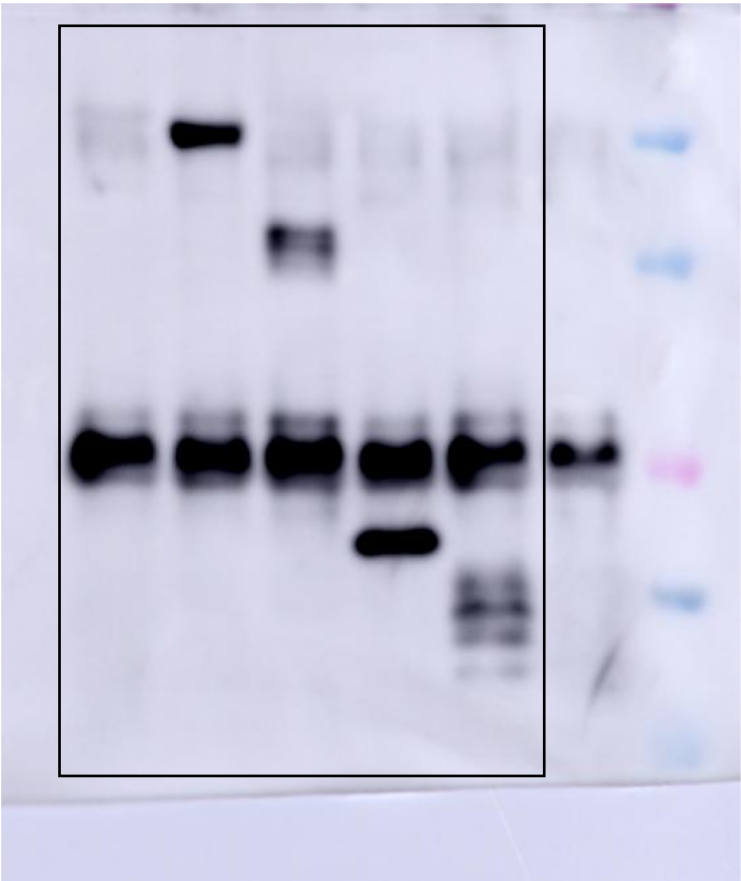

Flag

Supplement: Figure 6—source data 7. [file elife-89261-fig6-data7.zip › Figure 6 Source data 7.pdf]

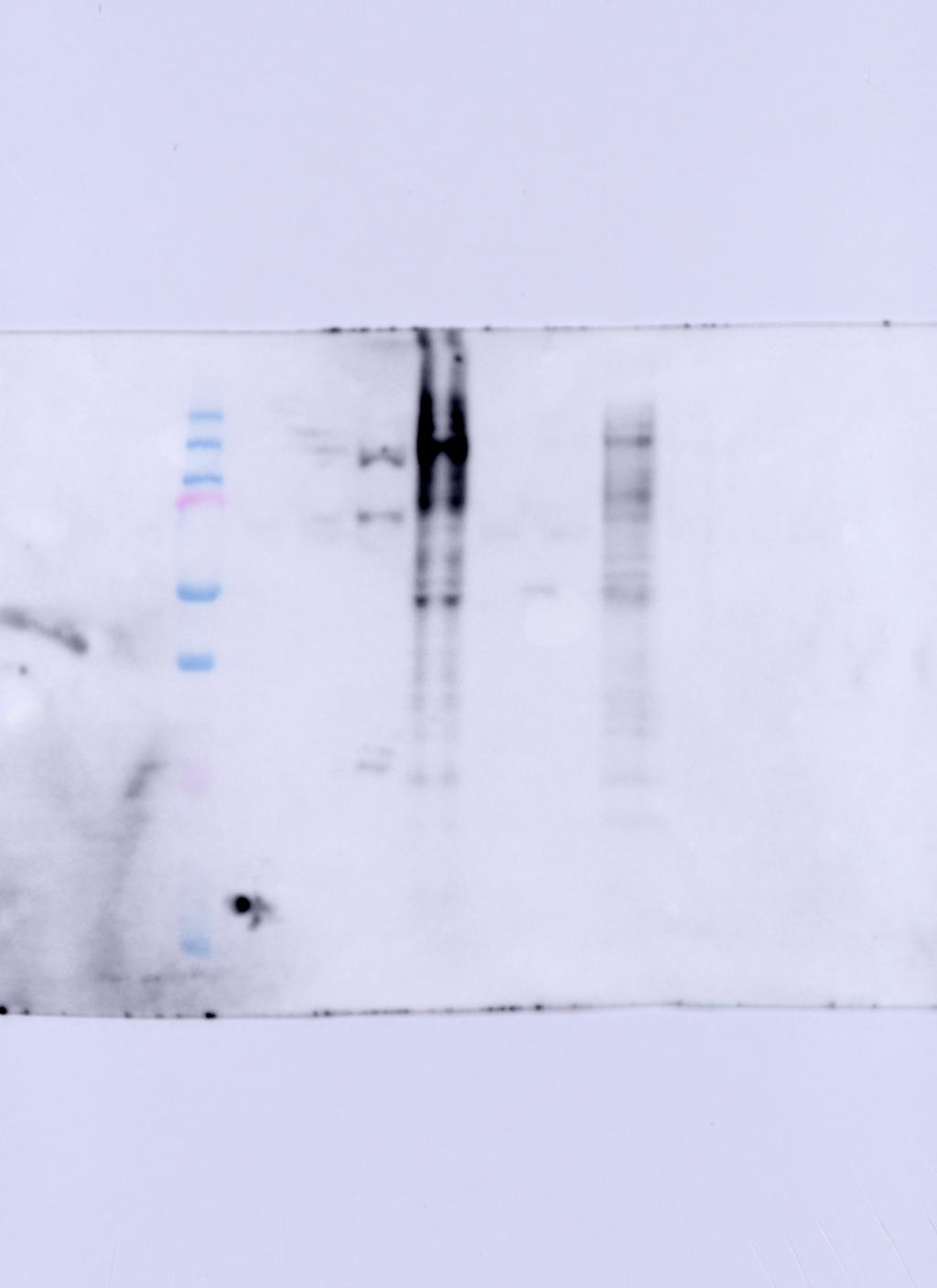

Supplement: Figure 6—figure supplement 1—source data 1. [file elife-89261-fig6-figsupp1-data1.zip › Figure 6-supplement figure 1-source data 1.tiff]

Fig6-figure supplement 1B

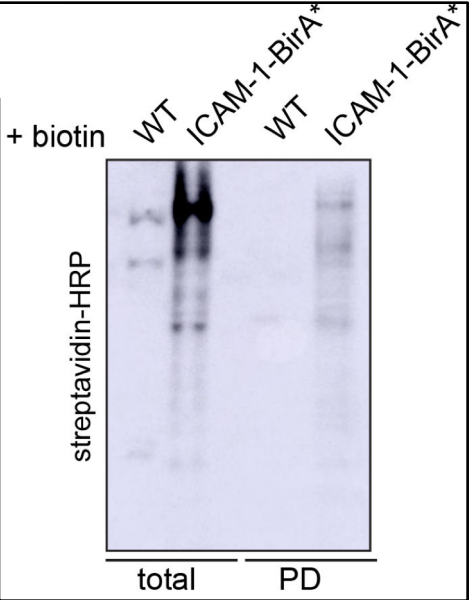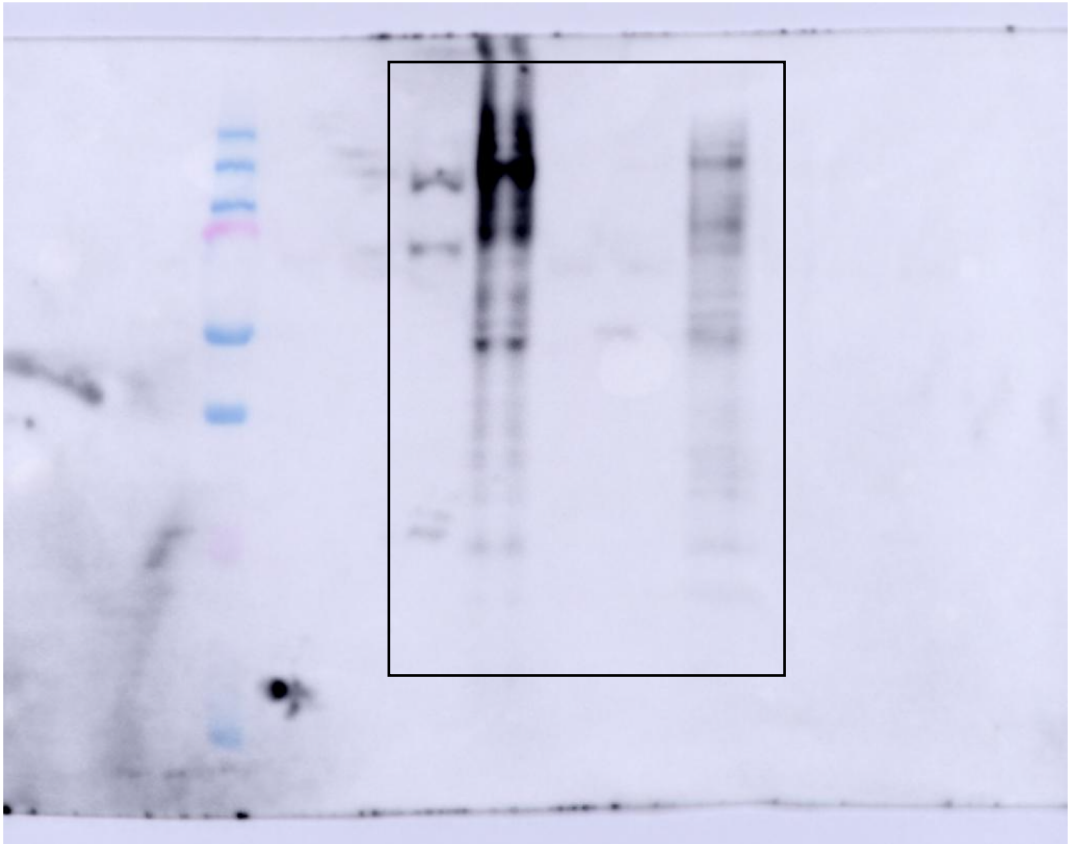

Supplement: Figure 6—figure supplement 1—source data 2. [file elife-89261-fig6-figsupp1-data2.zip › Figure 6-supplement figure 1-source data 2.pdf]

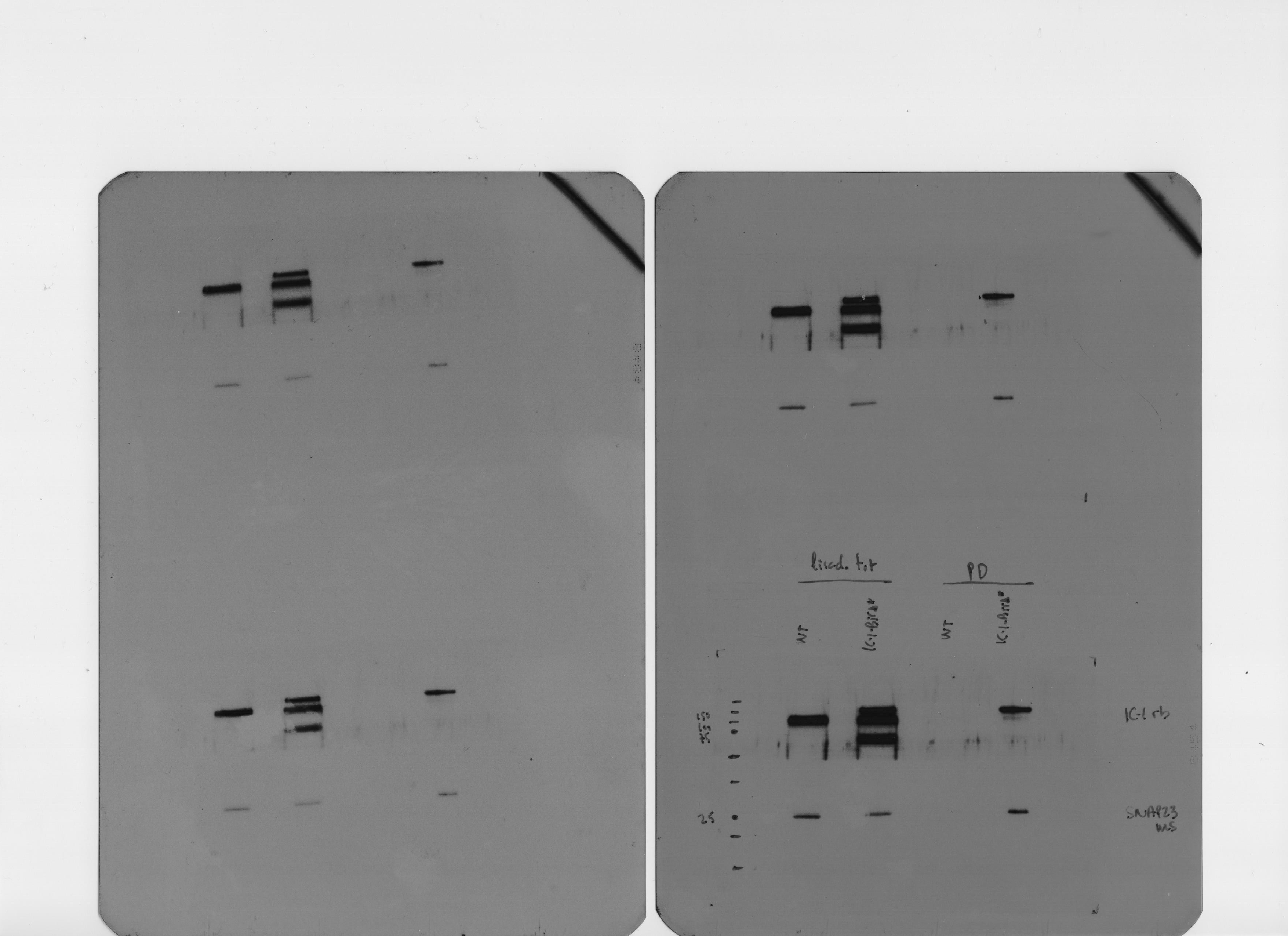

Supplement: Figure 6—figure supplement 1—source data 3. [file elife-89261-fig6-figsupp1-data3.zip › Figure 6-supplement figure 1-source data 3.tiff]

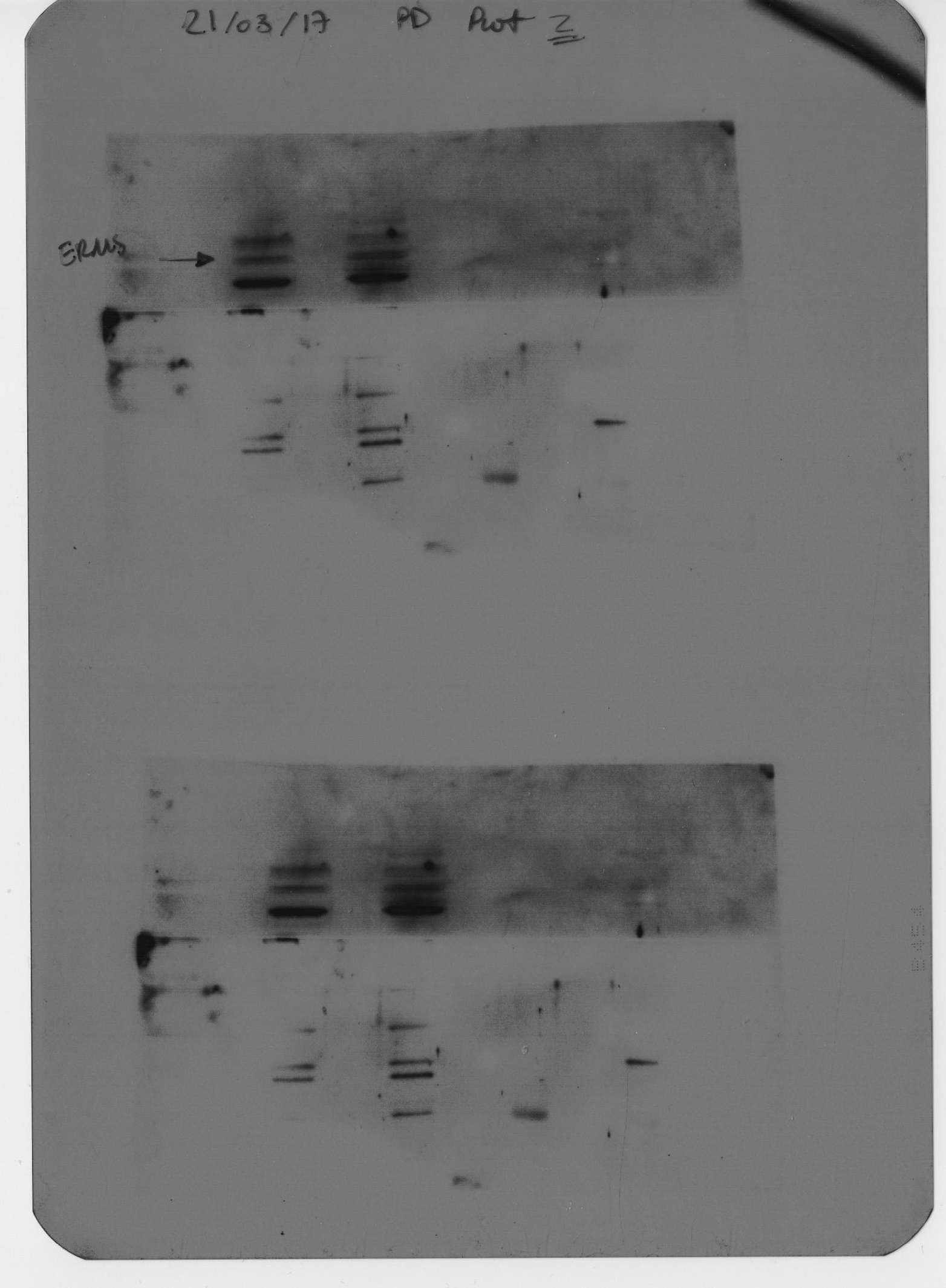

Supplement: Figure 6—figure supplement 1—source data 4. [file elife-89261-fig6-figsupp1-data4.zip › Figure 6-supplement figure 1-source data 4.tif]

Fig6-supplement figure 1D

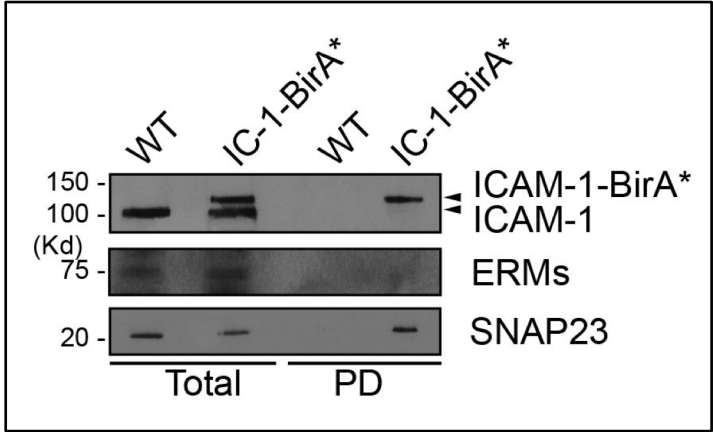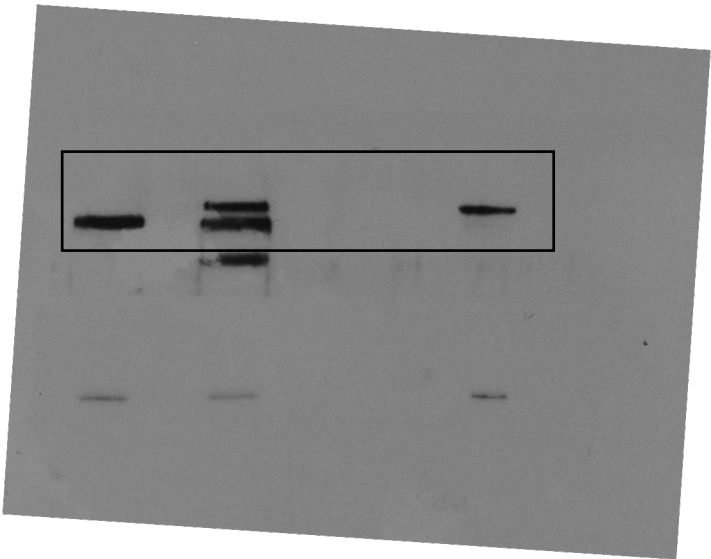

ICAM-1

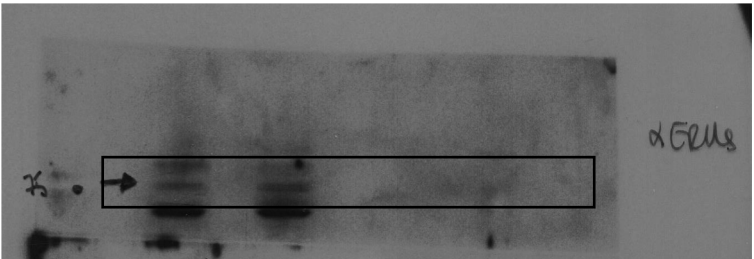

ERMs

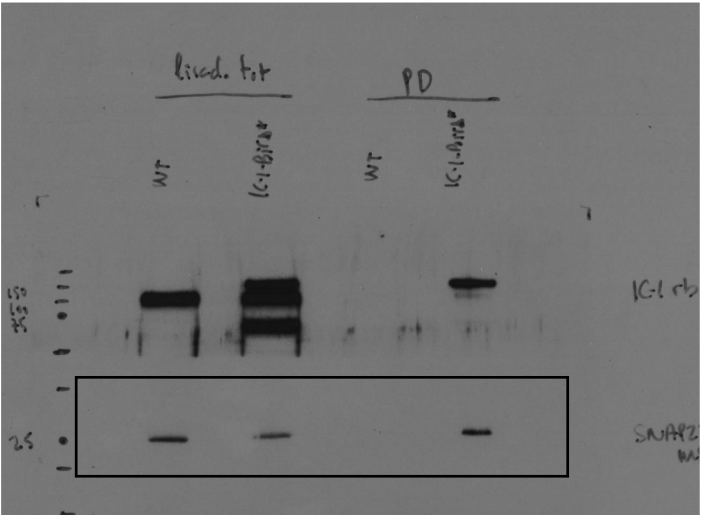

SNAP23

Supplement: Figure 6—figure supplement 1—source data 5. [file elife-89261-fig6-figsupp1-data5.zip › Figure 6-supplement figure 1-source data 5.pdf]

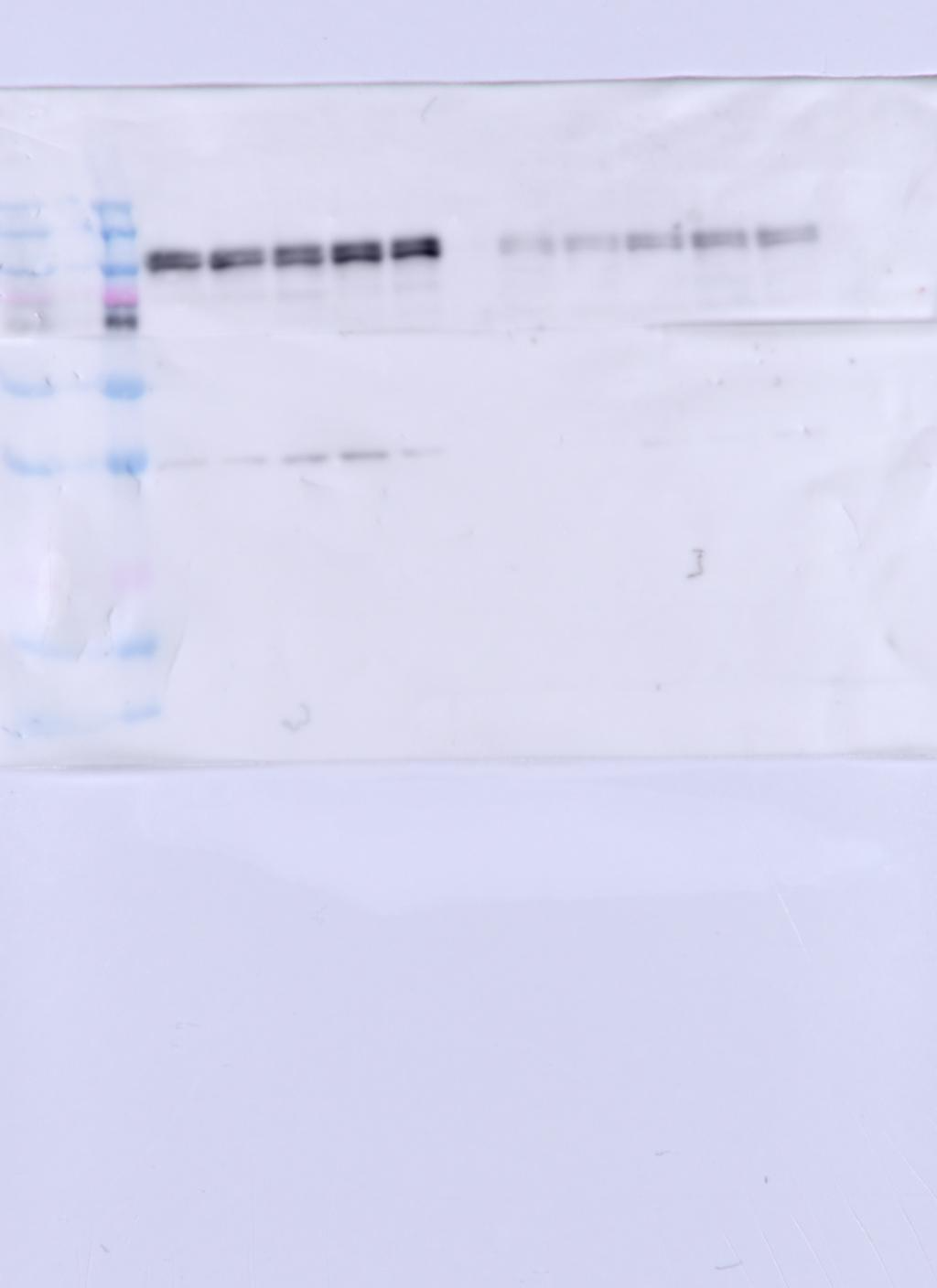

Supplement: Figure 6—figure supplement 1—source data 6. [file elife-89261-fig6-figsupp1-data6.zip › Figure 6-supplement figure 1-source data 6.tiff]

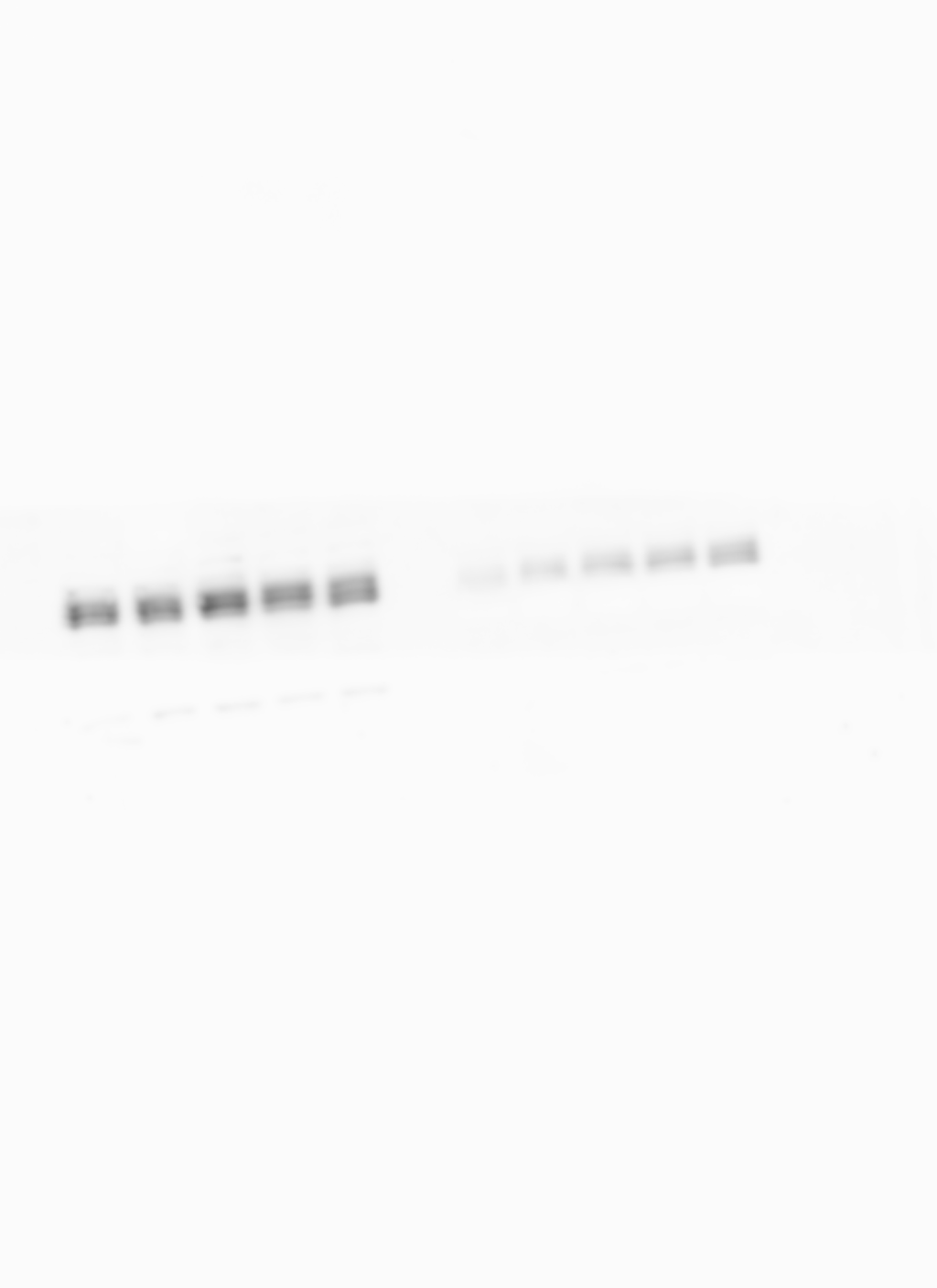

Supplement: Figure 6—figure supplement 1—source data 7. [file elife-89261-fig6-figsupp1-data7.zip › Figure 6-supplement figure 1-source data 7.tiff]

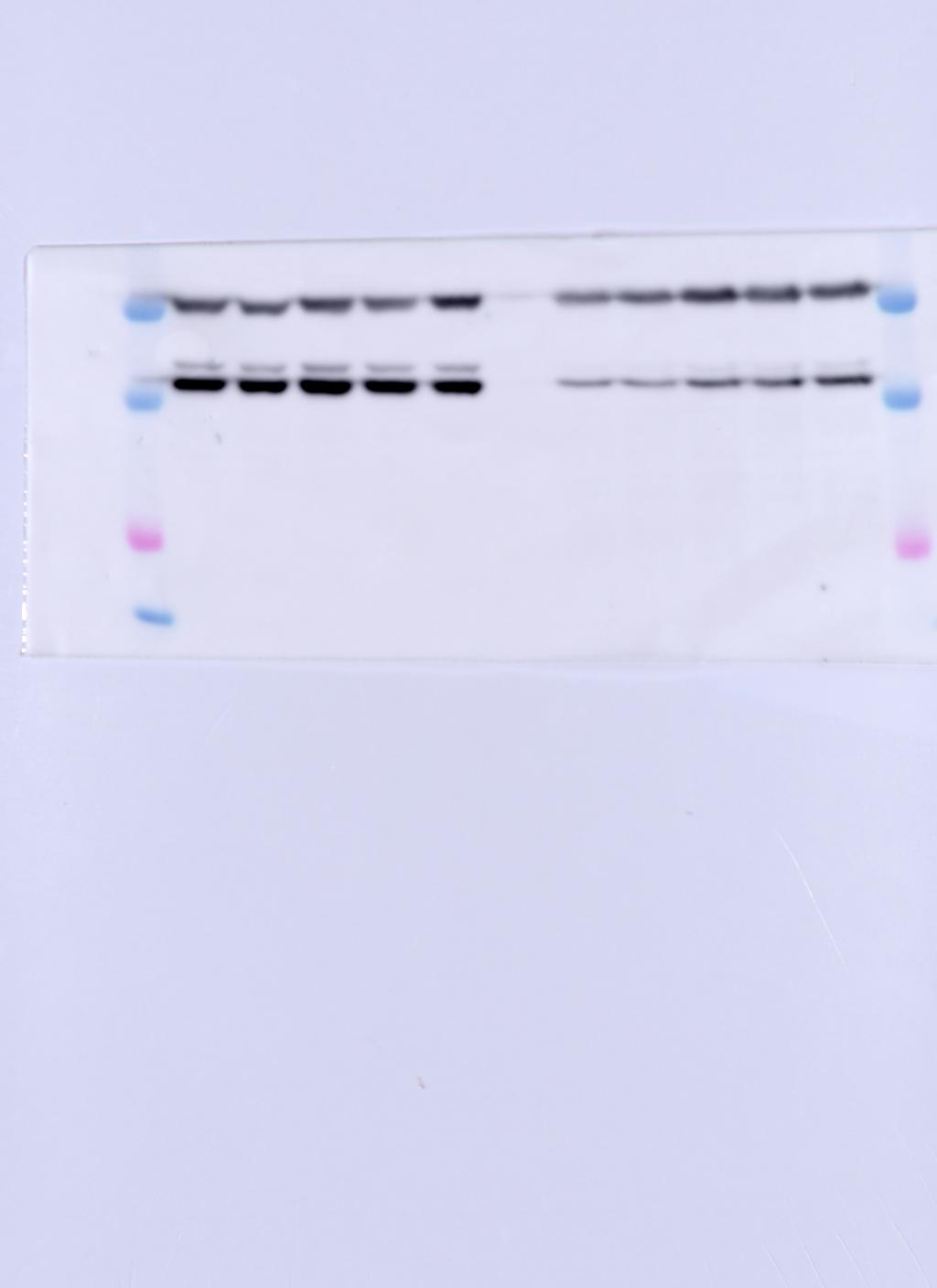

Supplement: Figure 6—figure supplement 1—source data 8. [file elife-89261-fig6-figsupp1-data8.zip › Figure 6-supplement figure 1-source data 8.tiff]

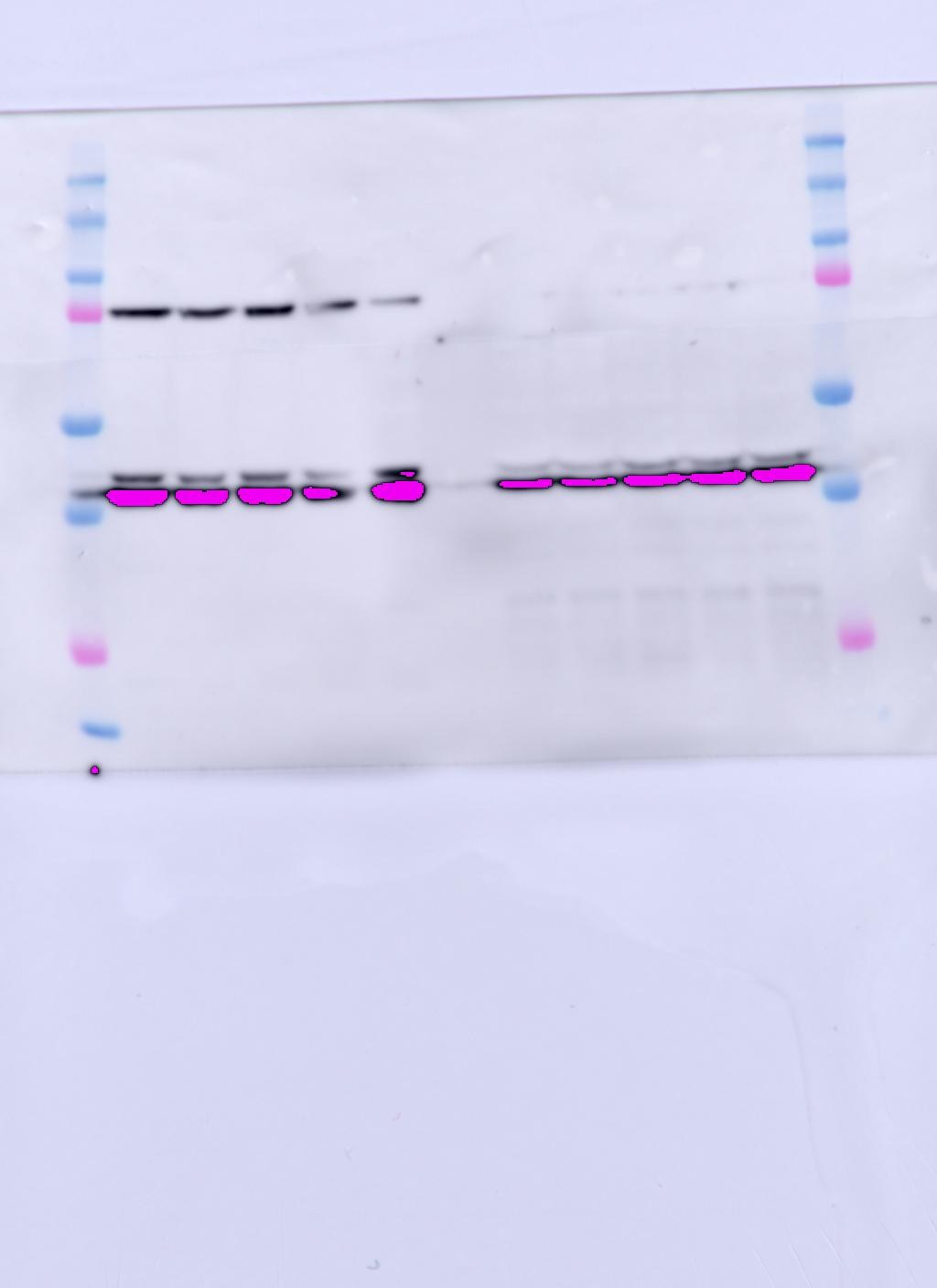

Supplement: Figure 6—figure supplement 1—source data 9. [file elife-89261-fig6-figsupp1-data9.zip › Figure 6-supplement figure 1-source data 9.tiff]

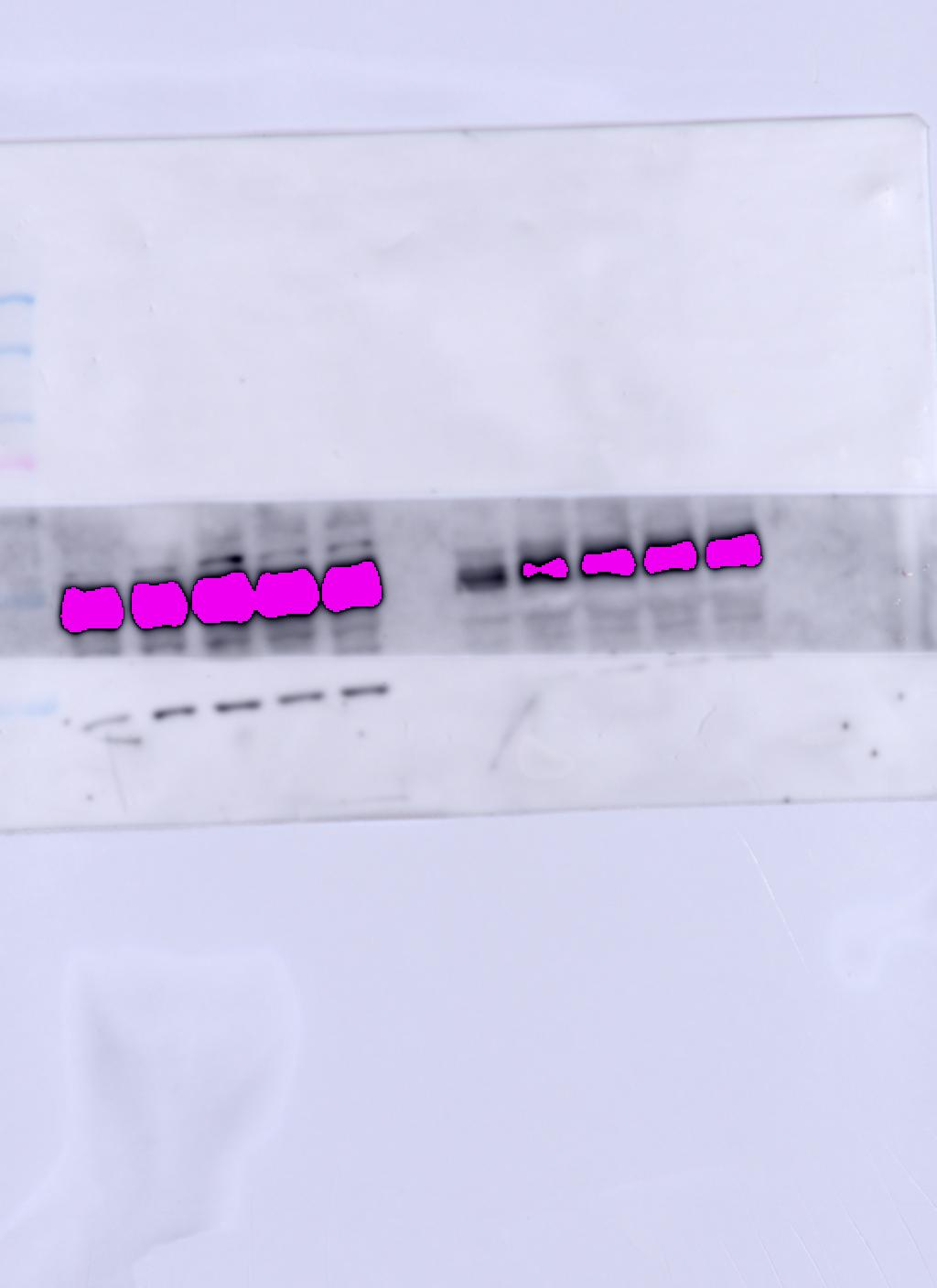

Supplement: Figure 6—figure supplement 1—source data 10. [file elife-89261-fig6-figsupp1-data10.zip › Figure 6-supplement figure 1-source data 10.tiff]

Fig6-supplement figure 1F

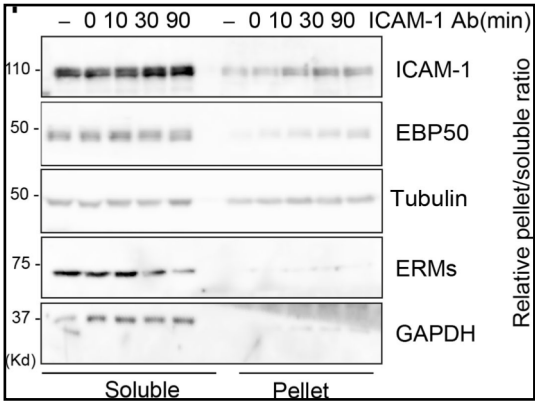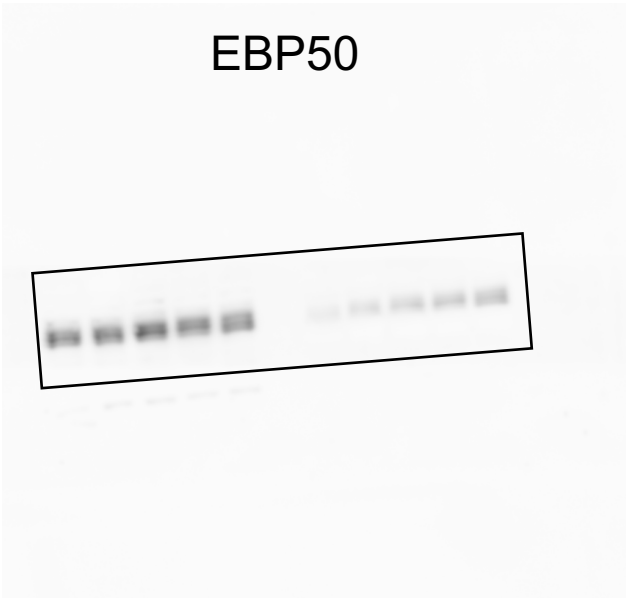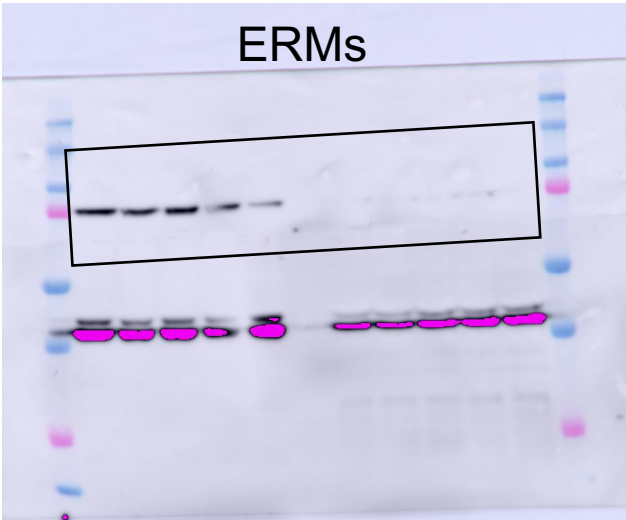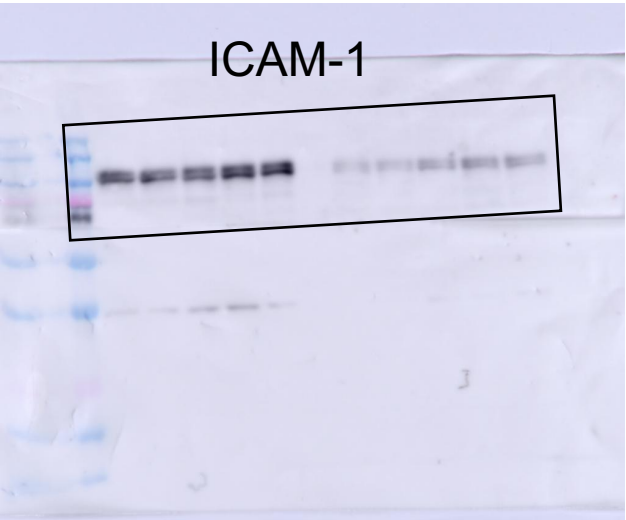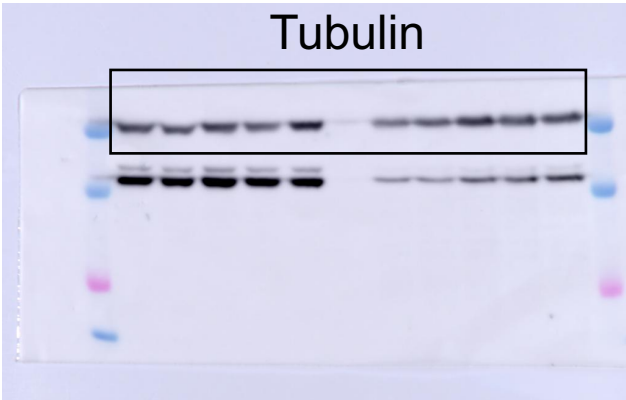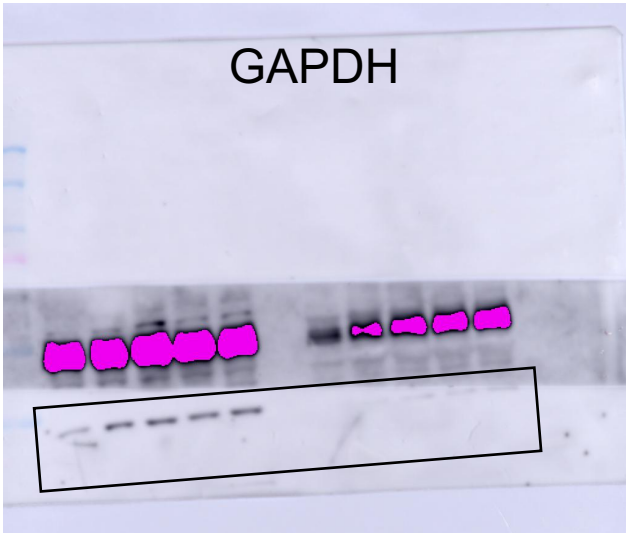

Supplement: Figure 6—figure supplement 1—source data 11. [file elife-89261-fig6-figsupp1-data11.zip › Figure 6-supplement figure 1-source data 12.pdf]

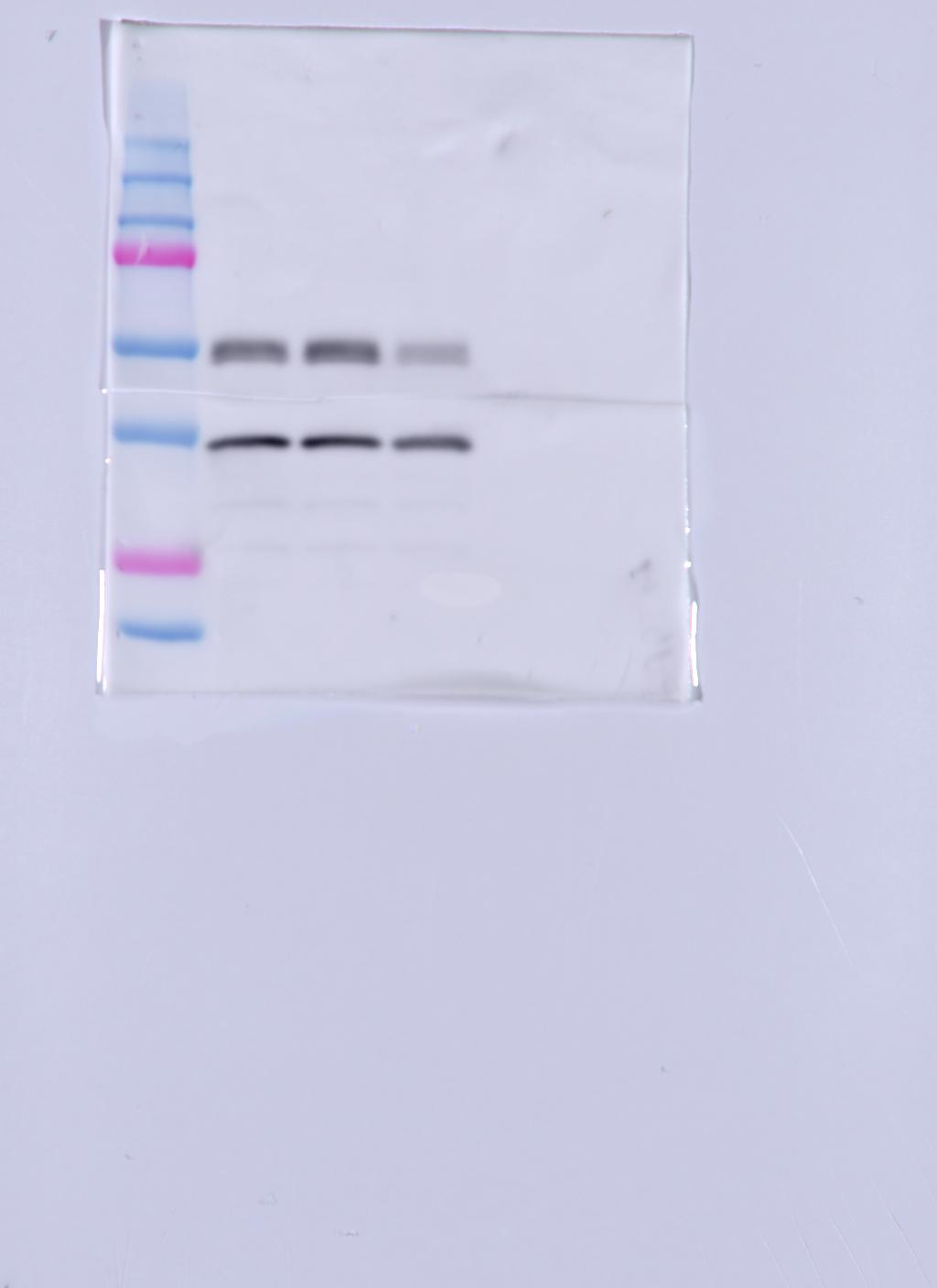

Supplement: Figure 7—source data 1. [file elife-89261-fig7-data1.zip › Figure 7 source data 1.tiff]

Fig. 7A

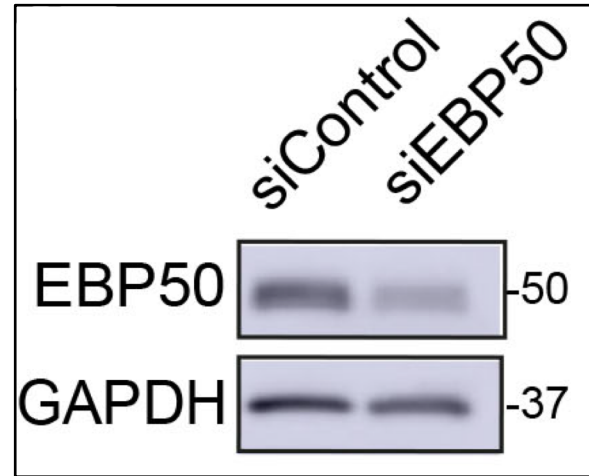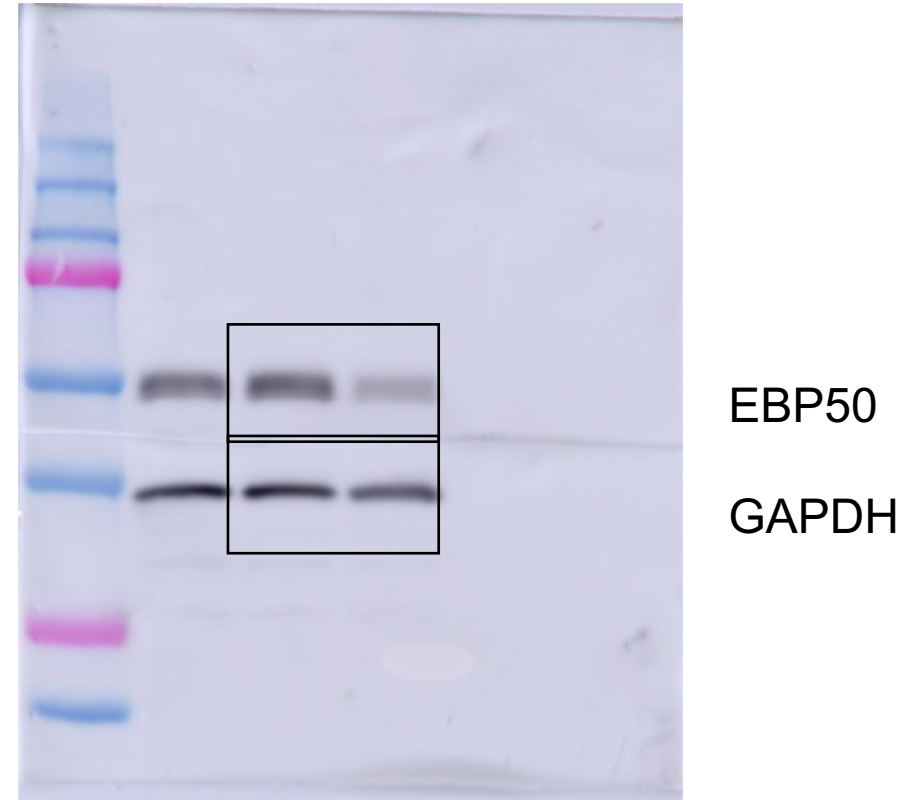

Supplement: Figure 7—source data 2. [file elife-89261-fig7-data2.zip › Figure 7 source data 2.pdf]

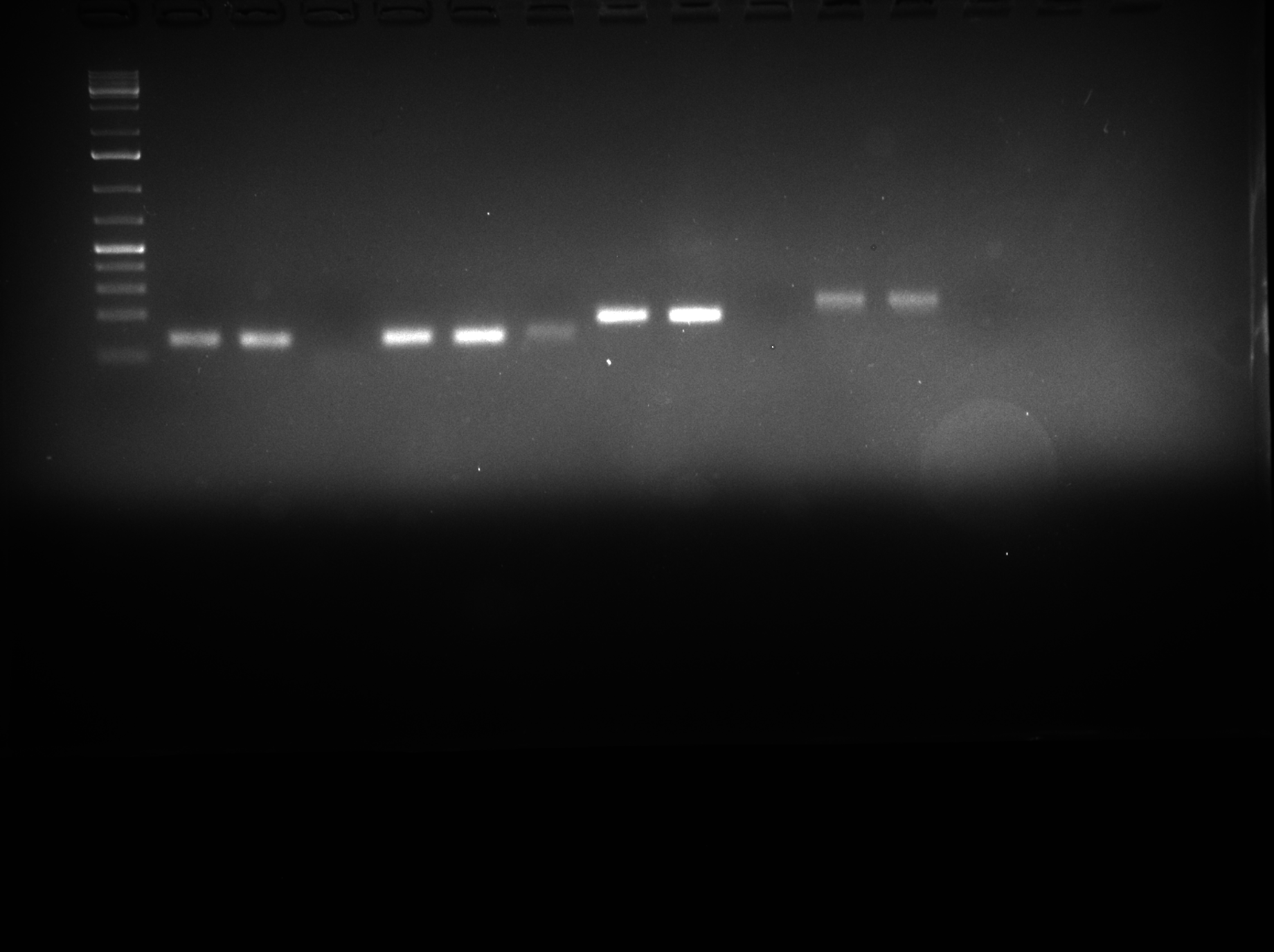

Supplement: Figure 8—figure supplement 1—source data 1. [file elife-89261-fig8-figsupp1-data1.zip › Figure 8-supplement figure 1-source data 1.tiff]

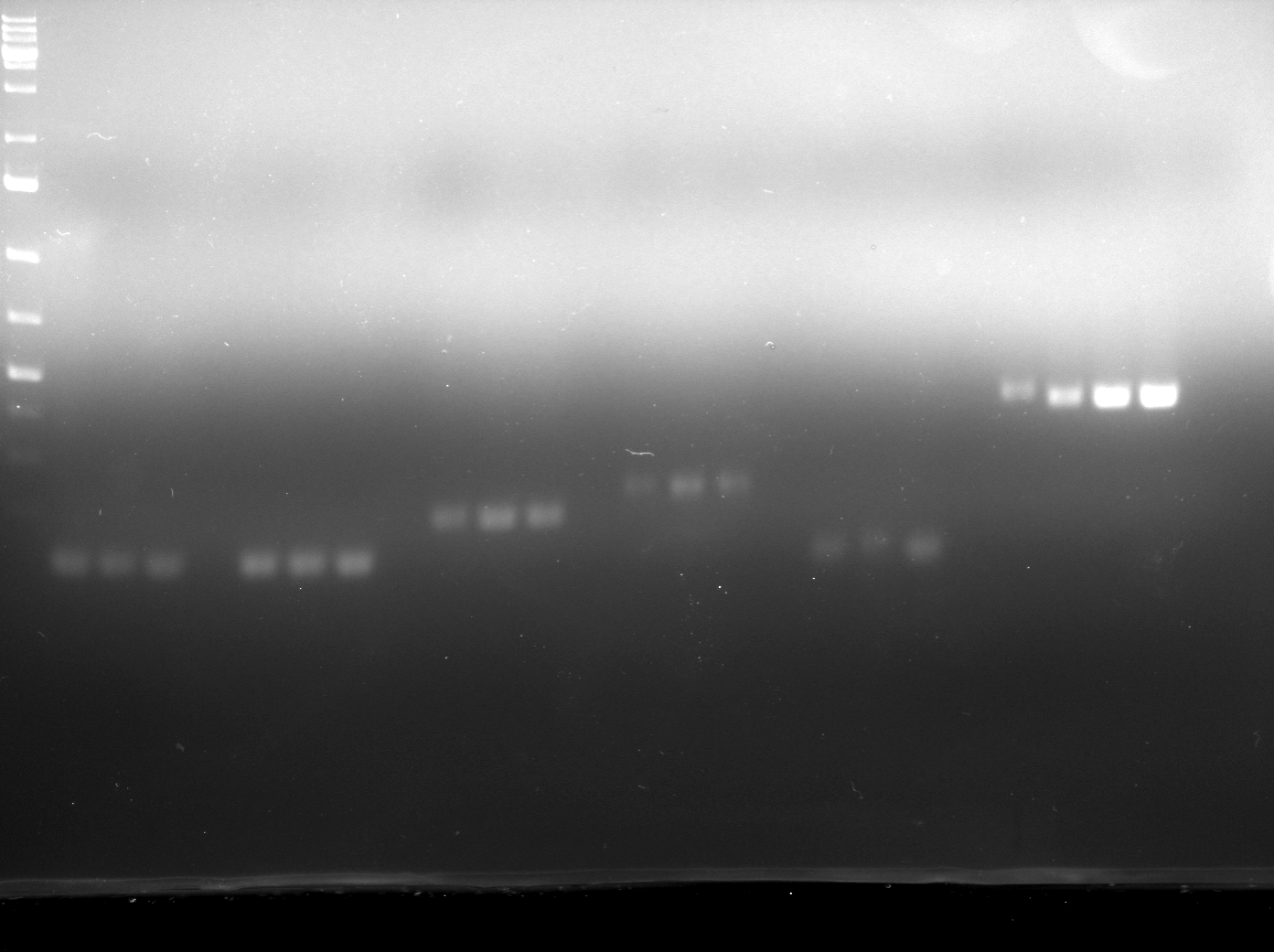

Supplement: Figure 8—figure supplement 1—source data 2. [file elife-89261-fig8-figsupp1-data2.zip › Figure 8-supplement figure 1-source data 2.tiff]

Fig8-supplement figure 1B

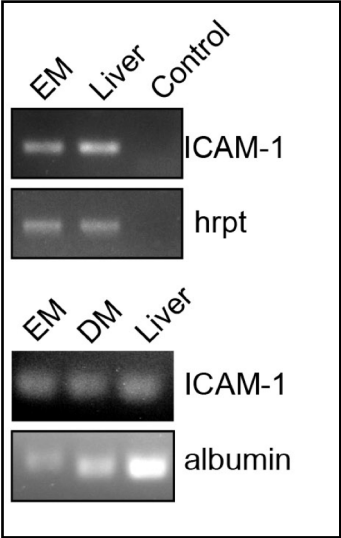

PCR ICAM-1 and hrpt

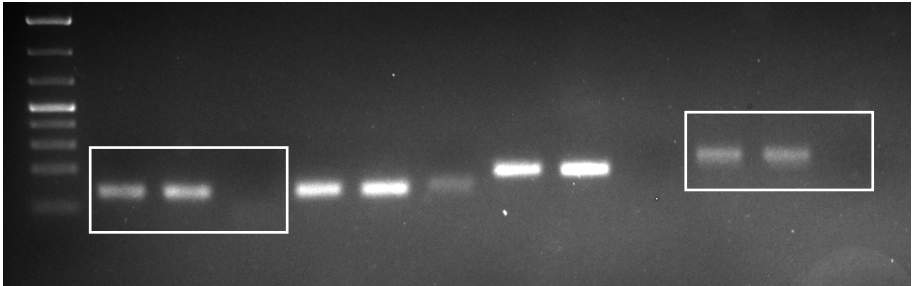

PCR ICAM-1 and albumin

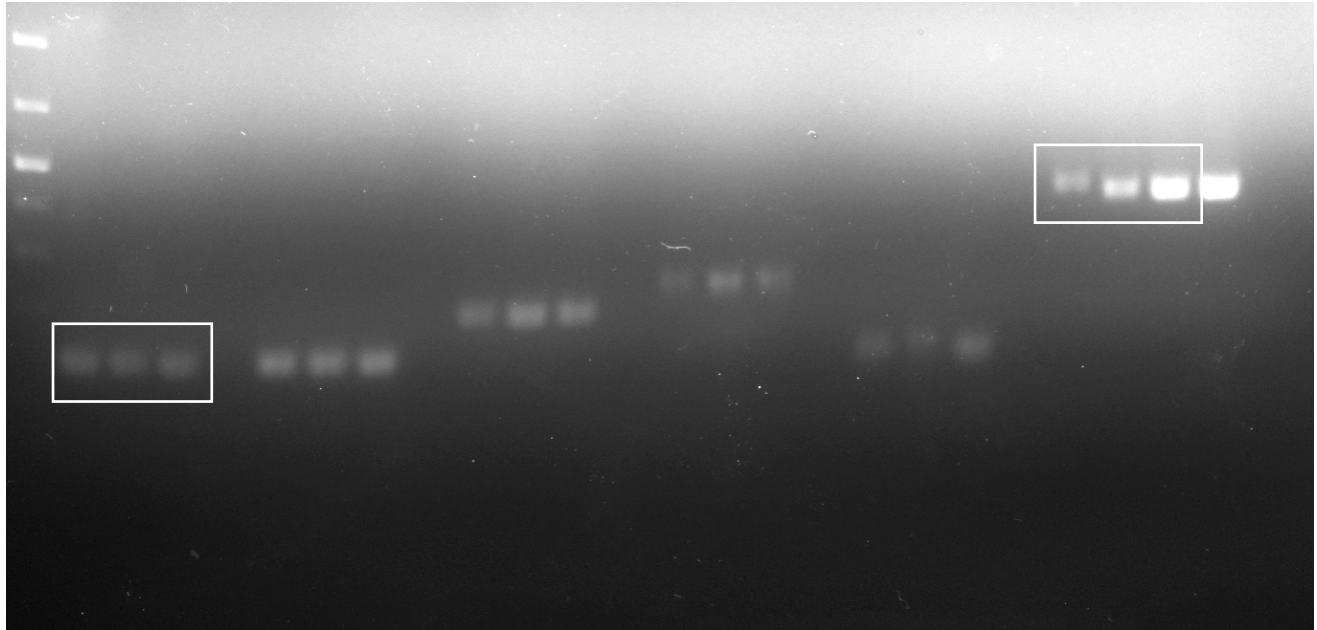

Supplement: Figure 8—figure supplement 1—source data 3. [file elife-89261-fig8-figsupp1-data3.zip › Figure 8-supplement figure 1-source data 3.pdf]
